# Biochemical and Structural Characterization of Neocartilage Formed by Mesenchymal Stem Cells in Alginate Hydrogels

**DOI:** 10.1371/journal.pone.0091662

**Published:** 2014-03-13

**Authors:** Magnus Ø. Olderøy, Magnus B. Lilledahl, Marianne Sandvold Beckwith, Jan Egil Melvik, Finn Reinholt, Pawel Sikorski, Jan E. Brinchmann

**Affiliations:** 1 The Norwegian Center for Stem Cell Research, Oslo University Hospital, Oslo, Norway; 2 Department of Physics, Norwegian University of Science and Technology, Trondheim, Norway; 3 NovaMatrix/FMC Biopolymer, Sandvika, Norway; 4 Department of Pathology, University of Oslo and Oslo University Hospital, Oslo, Norway; University of California, San Diego, United States of America

## Abstract

A popular approach to make neocartilage in vitro is to immobilize cells with chondrogenic potential in hydrogels. However, functional cartilage cannot be obtained by control of cells only, as function of cartilage is largely dictated by architecture of extracellular matrix (ECM). Therefore, characterization of the cells, coupled with structural and biochemical characterization of ECM, is essential in understanding neocartilage assembly to create functional implants in vitro. We focused on mesenchymal stem cells (MSC) immobilized in alginate hydrogels, and used immunohistochemistry (IHC) and gene expression analysis combined with advanced microscopy techniques to describe properties of cells and distribution and organization of the forming ECM. In particular, we used second harmonic generation (SHG) microscopy and focused ion beam/scanning electron microscopy (FIB/SEM) to study distribution and assembly of collagen. Samples with low cell seeding density (1e7 MSC/ml) showed type II collagen molecules distributed evenly through the hydrogel. However, SHG microscopy clearly indicated only pericellular localization of assembled fibrils. Their distribution was improved in hydrogels seeded with 5e7 MSC/ml. In those samples, FIB/SEM with nm resolution was used to visualize distribution of collagen fibrils in a three dimensional network extending from the pericellular region into the ECM. In addition, distribution of enzymes involved in procollagen processing were investigated in the alginate hydrogel by IHC. It was discovered that, at high cell seeding density, procollagen processing and fibril assembly was also occurring far away from the cell surface, indicating sufficient transport of procollagen and enzymes in the intercellular space. At lower cell seeding density, the concentration of enzymes involved in procollagen processing was presumably too low. FIB/SEM and SHG microscopy combined with IHC localization of specific proteins were shown to provide meaningful insight into ECM assembly of neocartilage, which will lead to better understanding of cartilage formation and development of new tissue engineering strategies.

## Introduction

Articular cartilage is a tissue consisting mainly of proteoglycans, type II collagen and water. The most abundant proteoglycan of articular cartilage is aggrecan, which due to high contents of glycosaminoglycan (GAG) side chains are negatively charged polyelectrolytes that create a natural swelling potential, constituting the main origin of the tissue’s compressive properties. The proteoglycans are anchored to a backbone of hyaluronic acid. This highly hydrated gel resides in a network of type II collagen fibrils, which provide tensile and shear mechanical properties. Chondrocytes reside in this functional tissue and maintain its structure, although it is known that the turnover of cartilage matrix is very slow. Due to the avascular and aneural nature and low cell numbers of articular cartilage, there is a limited capacity of tissue regeneration, which often leads to degenerative diseases upon injury. However, treatment commonly leads to the formation of fibrocartilage [1].

Tissue engineers are working to replace injured articular cartilage with tissue grown in the lab, where one popular approach is to culture chondrocytes or mesenchymal stem cells (MSC) in hydrogels to support the synthesis of new extracellular matrix (ECM) [2]–[11]. One of the main challenges using such approaches is the assembly of ECM proteins into functional structures, mimicking those of the natural tissue. It is not sufficient for the immobilized cells to merely produce the right combination of ECM proteins; they must be secreted, transported away from the cell surface, assemble into larger structures and interact with each other to recreate the fine-tuned mechanics of articular cartilage. One example is type II collagen, synthesized in the chondrocyte as type II procollagen, which is formed as a triple helical collagen domain terminated by globular N- and C-terminal propeptides [12]. These propeptides render the protein soluble and hinder spontaneous fibril formation in unwanted locations. The chondrocyte also produces propeptidases, e.g. ADAMTS-3 and BMP-1, which cleave off the N- and C-terminal propeptides [13], respectively, of type II procollagen. After cleavage, type II collagen can spontaneously assemble into fibrils, a process which is controlled also by small proteoglycans [14] and other collagens [12], [15]. The transport of these two enzymes and the collagen molecules before and after propeptide cleavage within the biomaterial matrix used for cell immobilization are therefore key regulators of tissue assembly and distribution of ECM structures as the tissue engineered cartilage develops. The dynamics of tissue assembly will vary greatly with type of hydrogel used to immobilize the cells, where electrostatic charges, polymer density, porosity, cell-polymer interactions and mechanical properties are important parameters. Further, engineered degradation of the hydrogel matrix has been used as a tool to accommodate the transport of ECM molecules and their assembly through the hydrogel [6], [7], [9], [16]–[19].

Alginates are polysaccharides of marine origin, which are composed of guluronic- and mannuronic acid in a block-wise fashion. Alginate hydrogels can rapidly be formed with most divalent cations, and Ca-alginate hydrogels may be formed at near-physiological conditions, which facilitates a gentle way of immobilizing human cells within the hydrogel. Interactions between alginate and cells are limited, but can be achieved by using alginates coupled to certain peptide sequences recognized by integrins in the cell membrane. Thus, cell-matrix interactions can be tailored for a given application. In tissue engineering of functional ECM, the alginate hydrogel can be dissolved gradually by incubation in solutions of reduced calcium concentration, or polymer degradation may be tailored by using oxidized alginates with a bimodal molecular weight distribution [20]. Further, it is possible to control the gelling time by using a self-gelling approach to the hydrogel formation. The self-gelling principle utilizes lyophilized and milled Ca-alginate particles with a controlled size distribution in the µm range. Dispersion of such particles in a Na-alginate solution with a controlled NaCl concentration will lead to controlled release of Ca^2+^ from the particles, which will lead to cross-linking of the alginate solution, and thus hydrogel formation [21]. By these means, human cells may be immobilized in a gentle way at near physiological conditions in alginate hydrogels with any wanted shape within minutes. Such an approach was used in the present work.

In this study, we differentiated MSC into chondrocytes in an alginate hydrogel matrix, and aimed to investigate the assembly of functional collagen structures. We developed a combination of methods that can provide structural, biochemical and functional characteristics of tissue engineered cartilage. First, we characterized the distribution of type II collagen using fluorescence immunohistochemistry (IHC). Then we probed the forming tissue for fibrillar collagen assemblies, using second harmonic generation (SHG) microscopy. Further, to characterize the three-dimensional (3D) network of fibrillar collagens close to a cell residing in the alginate matrix, we used so-called FIB/SEM tomography, where focused ion beam (FIB) milling of the sample surface and scanning electron microscopy (SEM) are combined to obtain 3D data of the formed matrix, with nanometer resolution in all three spatial dimensions. This was not done for all sample types and time points, but was used as a method to verify and to help interpret data obtained using other techniques. Finally, the enzymes involved in type II procollagen processing were addressed using fluorescence IHC and assembly mechanisms that can potentially be taken advantage of are discussed. These techniques in combination constitute a powerful tool-box to characterize the formation of functional ECM structures in tissue engineered cartilage, and clearly show that there are unresolved challenges regarding tissue engineering based on ECM-producing cells immobilized in hydrogels.

## Materials and Methods

All the chemicals were purchased from Sigma-Aldrich (St. Louis, MO) unless otherwise stated.

### Ethics Statement

The study was approved by the Regional Committee for Medical Research Ethics, Southern Norway, Section A. Participants provided their written informed consent to participate in the study.

### Cell Isolation and Expansion

40–50 ml of bone marrow was aspirated from the iliac crest of one healthy voluntary donor, and the mononuclear cells were isolated from the aspirate using density gradient centrifugation (Lymphoprep, Fresenius Kabi, Oslo, Norway). The cells were seeded in 175 cm^2^ flasks (Nunc, Roskilde, Denmark) and cultured in Dulbecco’s Modified Eagle Medium:Nutrient Mixture F-12 containing GlutaMAX (DMEM/F-12 GlutaMAX, Gibco, Paisley, UK), supplemented with 10% pooled human platelet lysate plasma (PLP), 1% penicillin/streptomycin and 1.5 µg/ml amphotericin B. After 24–48 hours, non-adherent cells were removed by medium change and the remaining cells were expanded using the medium described above, but without amphotericin B.

### Cell Immobilization and Differentiation in Alginate Hydrogels

Cells were immobilized in alginate hydrogels shaped as discs (height 2 mm, diameter 5 mm) by using a self-gelling alginate technology (NovaMatrix, Sandvika, Norway). Insoluble particles (25–45 µm) of Ca-alginate (oligomeric alginate (G-blocks) cross-linked with calcium) were dispersed in 4.6% (w/v) mannitol to a concentration of 1.6% (w/v). Ultra pure, low viscosity, high G Na-alginate (PRONOVA UP LVG, NovaMatrix) was dissolved in 4.6% (w/v) mannitol to a concentration of 1.6% (w/v) and sterile filtered through a 0.2 µm syringe filter. MSC were lifted with trypsin/EDTA, centrifuged to a pellet and washed with 4.6% (w/v) mannitol to remove all traces of calcium and non-gelling ions. Then, the final cell pellet was dispersed gently in the Na-alginate solution, and mixed in equal volumes with the Ca-alginate suspension using a pipette. This mixture was distributed into custom-made molds to make 45 µl hydrogels with a final cell density between 10–50×10^6 ^ml^−1^. The hydrogels were allowed to set for 15 minutes at room temperature, before they were gently washed with 50 mM CaCl_2_ in 0.56% (w/v) NaCl for 5 minutes. The hydrogels were transferred into 24-well plates (Costar, Corning, NY), where each well contained 1 ml of chondrogenic differentiation medium consisting of DMEM/F12 GlutaMAX, supplemented with 1% penicillin/streptomycin, 1 mM sodium pyruvate (Gibco, Paisley, UK), 0.1 mM ascorbic acid-2-phosphate, 0.1 mM dexamethasone, 1% ITS (insulin 25 mg/ml, transferrin 25 mg/ml, and sodium selenite 25 ng/ml), 1.25 mg/ml human serum albumin (Octapharma, Hurdal, Norway), 500 ng/ml bone morphogenic protein-2 (InductOs, Wyeth, Taplow, UK), and 10 ng/ml recombinant human transforming growth factor-β1 (TGFβ1, R&D Systems, Minneapolis, MN). This medium was changed thrice a week. After 10 days of chondrogenic differentiation, the hydrogels were split in two groups; one continuing with the chondrogenic medium described above (containing 1.05 mM calcium) and the other receiving a similar medium, but with only 0.6 mM calcium to reduce the hydrogel stability. The samples are summarized in Table 1.

**Table 1:** Samples characterized in this study differed in cell seeding density (δ) and calcium concentration ([Ca^2+^]) in the differentiation medium.

Sample name	δ (×10^6^ml^−1^)	[Ca^2+^] (mM)
10-A	10	0.6
10-B	10	1.05
25-A	25	0.6
25-B	25	1.05
50-A	50	0.6
50-B	50	1.05

All samples were cultured with 1.05 mM calcium until day 10, and then switched to the given concentrations.

### Quantitative Real-time Reverse Transcription Polymerase Chain Reaction (RT qPCR)

Total RNA was isolated from MSC in alginate hydrogels using the RNeasy kit (Qiagen, Valencia, CA), after first grinding the hydrogels in Eppendorf tubes with corresponding pestles while frozen in liquid N_2_ and further grinding into a fine powder using QiaShredder columns (Qiagen). Quantification of the purified RNA was done using a NanoDrop ND-1000 spectrophotometer (Nanodrop Technologies, Wilmington, DE). RNA was treated with DNase I (Ambion, Austin, TX) and reverse transcribed into cDNA according to the manufacturer’s protocol (Applied Biosystems, Abingdon, UK). Polymerase chain reaction was used to probe cDNA samples for relevant genes, using primers from Applied Biosystems (see Table 2).

**Table 2:** PCR primers and IHC antibodies used to probe respective proteins.

Protein	PCR primer	Antibodies for IHC		
		Designation (conc.)	Isotype	Company
Type I collagen	Hs00164004 m1	–	–	–
Type II collagen	Hs00264051 m1	II-4C11 (0.83 µg/ml)	Mouse IgG1	MP Biomedicals
Type II procollagen	–	ab17771 (1.00 µg/ml)	Mouse IgG1	Abcam
Type VI collagen	–	5C6-c (1.96 µg/ml)	Mouse IgG1	DSHB
Type X collagen	Hs00166657 m1	–	–	–
Aggrecan	Hs00202971 m1	969D4D11 (4.55 µg/ml)	Mouse IgG1	BioSource
ADAMTS-2	Hs01029111 m1	ab125226 (0.25 µg/ml)	Rabbit IgG	Abcam
ADAMTS-3	Hs00610744 m1	ab84795 (0.25 µg/ml)	Rabbit IgG	Abcam
BMP-1	Hs00241807 m1	ab118520 (1.00 µg/ml)	Rabbit IgG	Abcam
MMP-13	Hs00233992 m1	–	–	–
GAPDH	Hs99999905 m1	–	–	–
Isotype control	–	ab27478	Rabbit IgG	Abcam

### Fluorescence Immunohistochemistry

Samples were fixed in 4% paraformaldehyde and embedded in paraffin before being cut and mounted on polysine glass slides. Those sections were deparaffinized and rehydrated using standard laboratory procedures, then post fixed in 4% paraformaldehyde in phosphate buffered saline for 10 minutes. Heat mediated antigen retrieval was carried out for 20 min in 0.05% citraconic anhydride buffer pH 7.4 for all stainings except for BMP-1 where 10 mM citrate buffer pH 6.0 was used. Then, primary antibodies in 1.25% bovine serum albumin (BSA) were applied (according to Table 2) and incubated overnight at 4°C. Negative controls were made omitting primary antibody, while for ADAMTS-2, ADAMTS-3 and BMP-1, isotype controls were used. After rinsing, secondary antibodies in 12.5% BSA were applied at room temperature for 1.5 hours. The secondary antibodies used were goat anti-rabbit IgG conjugated to Alexa 488 (Invitrogen, Carsbad, CA) and donkey anti-mouse IgG conjugated to Cy3 (Jackson Immuno Research Europe, Newmarket, UK), used at 5.0 and 1.4 µg/ml, respectively. The stained sections were mounted with prolong gold antifade (Invitrogen), containing DAPI for nuclear stain. Imaging was done using an upright Nikon Eclipse E600 microscope equipped with an Olympus ColorView III camera.

### Second Harmonic Generation Microscopy

SHG microscopy was performed on a Leica SP8 confocal imaging system equipped with a Coherent Vision S femtosecond laser for SHG with collagen and for two-photon excitation of DAPI. Collagen was excited at 900 nm and SHG detected using a non-descanned transmitted light detector with a 435–455 nm bandpass filter. DAPI was excited at 800 nm and fluorescence was collected with an internal photo multiplicator tube detector in the confocal scanner with bandpass filter set to 410–450 nm. Images were collected with a 25x objective with numerical aperture of 0.9.

SHG depends quadratically on the molecular concentration, which makes it difficult to provide useful structural information from high and low collagen density regions in the same image, using the same settings. In order to overcome this, we processed the images non-linearly as follows, finding the new intensity *I*

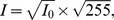
where *I_0_* is the original pixel intensity in the range 0–255. Thereby, *I* will be in the range 0–255, and the lower intensities will be enhanced relatively more than the higher values.

### Transmission Electron Microscopy

Samples were fixed in 2.5% (v/v) glutaraldehyde, 2% (w/v) paraformaldehyde in 0.1 M sodium cacodylate buffer for 24 hours, then post-fixed in 2% osmium tetroxide at 4°C for two hours. Dehydration was done gradually to ethanol with subsequent staining in 2% uranyl acetate and replacement of solvent with acetone. Acetone was gradually replaced with Epon, which was finally cured over night at 60°C. Ultrathin sections (80 nm) were cut and imaged using a TEM in bright field mode.

### Focused Ion Beam/scanning Electron Microscopy

After six weeks of chondrogenic differentiation in alginate, one sample was cut into smaller (≈0.5 mm) pieces, which were fixed for 3 hours at room temperature in 2.5% (v/v) glutaraldehyde, 2% (w/v) paraformaldehyde in 0.1 M sodium cacodylate buffer. Samples were then washed twice in 0.1 M sodium cacodylate buffer before staining, which was done using a protocol based on two previous papers [22], [23]. First, samples were submerged in 1.5% potassium ferrocyanide with 1% osmium tetroxide for 20 hours at room temperature, and washed for 3×10 min in 0.1 M sodium cacodylate buffer. Next, 1% tannic acid was applied for one hour, followed by rinsing 3×10 min in distilled water. Finally, samples were submerged in 1% uranyl acetate for two hours, and washed 3×10 min in distilled water. The stained samples were dehydrated in ethanol, which was replaced with acetone before acetone was gradually replaced with Epon. The resin was cured over night at 60°C. The sample-containing plastic block was cut with a hack-saw until sufficiently small size, before the front- and top faces were trimmed with a microtome and razor blade, respectively. The blocks were then glued on top of a standard aluminium sample stub using carbon tape. Subsequently, the samples were sputter coated (Cressington model 208 HR) twice at different angles, each time with a 20 nm thick layer of platinum/palladium. In the FIB/SEM instrument (FEI Helios NanoLab DualBeam), the front face was milled with the ion beam at 2.7 nA beam current until a cell with its peri- and extracellular matrix was located. Then, a protective platinum layer of 1.2 µm was deposited on the top face of the volume of interest (VOI) using the gas injection system in the FIB/SEM instrument. A fiduciary marker (X) was milled in this layer in order to support the automatic positioning done during FIB/SEM processing (see Figure S1). To avoid redeposition of material and shadowing during imaging, trenches of about 6 µm width were milled on both sides of the VOI with an ion beam current of 6.5 nA. Finally, the front surface was polished with the FIB, starting at 2.7 nA and ending at 0.44 nA beam current. Proper polishing is crucial to avoid curtaining artefacts during the experiment. 22 nm thick sections were milled with the FIB at a current of 0.92 nA, and SEM images were captured in immersion mode using the through lens detector in secondary electron mode. The electron beam acceleration voltage was set to 5 kV, the beam current to 0.69 nA and the dwell time to 10 µs during imaging. FIB milling and image acquisition were repeated sequentially using an automated procedure (Auto Slice and View G2, FEI) until approximately 300 images had been captured.

The captured data set was adjusted for drift using the StackReg plugin in ImageJ, with the transformation set to translation. Contrast and brightness were adjusted to correct for a decrease in contrast over the 300 images. The images were processed using the Gaussian Blur filter (Radius = 0.90) before Auto Local Threshold (Method = MidGrey, Radius = 30, Parameter 1 =  −10) was applied, following Gaussian Blur filter (Radius = 0.90). The thresholded image series was then reconstructed using the 3D Viewer plugin, with a resampling factor of 1. For the animations visualizing a sliding volume, custom scripts were made using the ImageJ Macro language.

## Results

First, chondrogenic differentiation was verified at mRNA and protein levels, and these samples were then used to study the assembly of ECM architecture. We followed the samples for six weeks and studied the various parameters at time points 0, 3 and 6 weeks. Finally, some proteins central for matrix assembly were studied in the context of spatial and temporal formation of tissue structures in the used alginate hydrogels.

### RT qPCR

**Figure 1:**
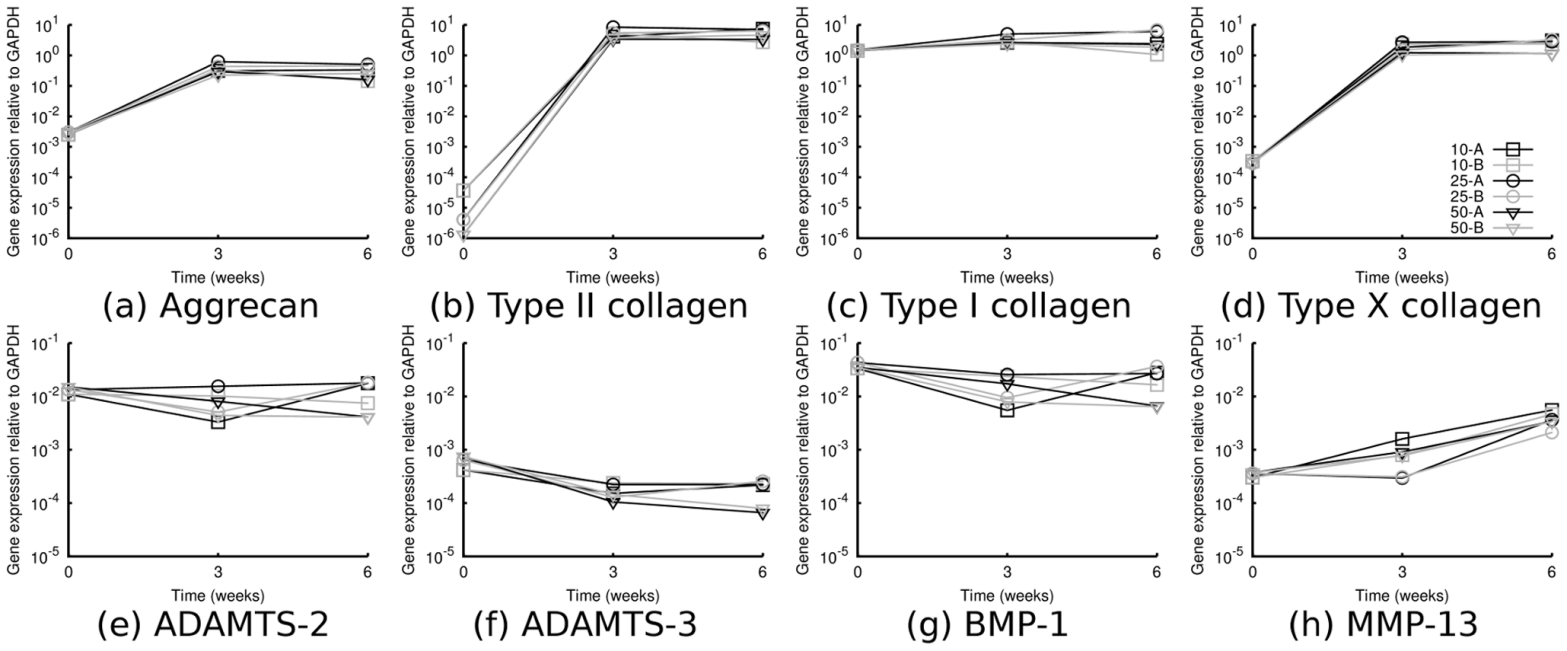
mRNA expression levels relative to GAPDH of selected genes relevant to chondrogenic differentiation, measured by RT qPCR. Structural ECM proteins (a–d) and enzymes involved in collagen processing and degradation (e–h).

Chondrogenic differentiation of MSCs in alginate hydrogels was verified by RT qPCR, with gene expression of central genes relative to GAPDH shown in Figure 1. Among the structural proteins in Figure 1 (a–d), all the used conditions resulted in upregulation of aggrecan and type II collagen, regardless of calcium concentration in the medium (0.6 or 1.05 mM) and cell seeding density (10, 25 or 50×10^6^ ml^−1^). In comparison, type I collagen mRNA levels were relatively unchanged, while type X collagen was upregulated markedly, indicating commonly observed chondrocyte hypertrophy. In Figure 1 (e–h), mRNA levels of selected enzymes with important functions in procollagen processing and collagen fibril degradation are shown. ADAMTS-2, ADAMTS-3 and BMP-1 were all detected and decreased slightly during the course of chondrogenic differentiation, while MMP-13 increased.

### Fluorescence IHC

**Figure 2:**
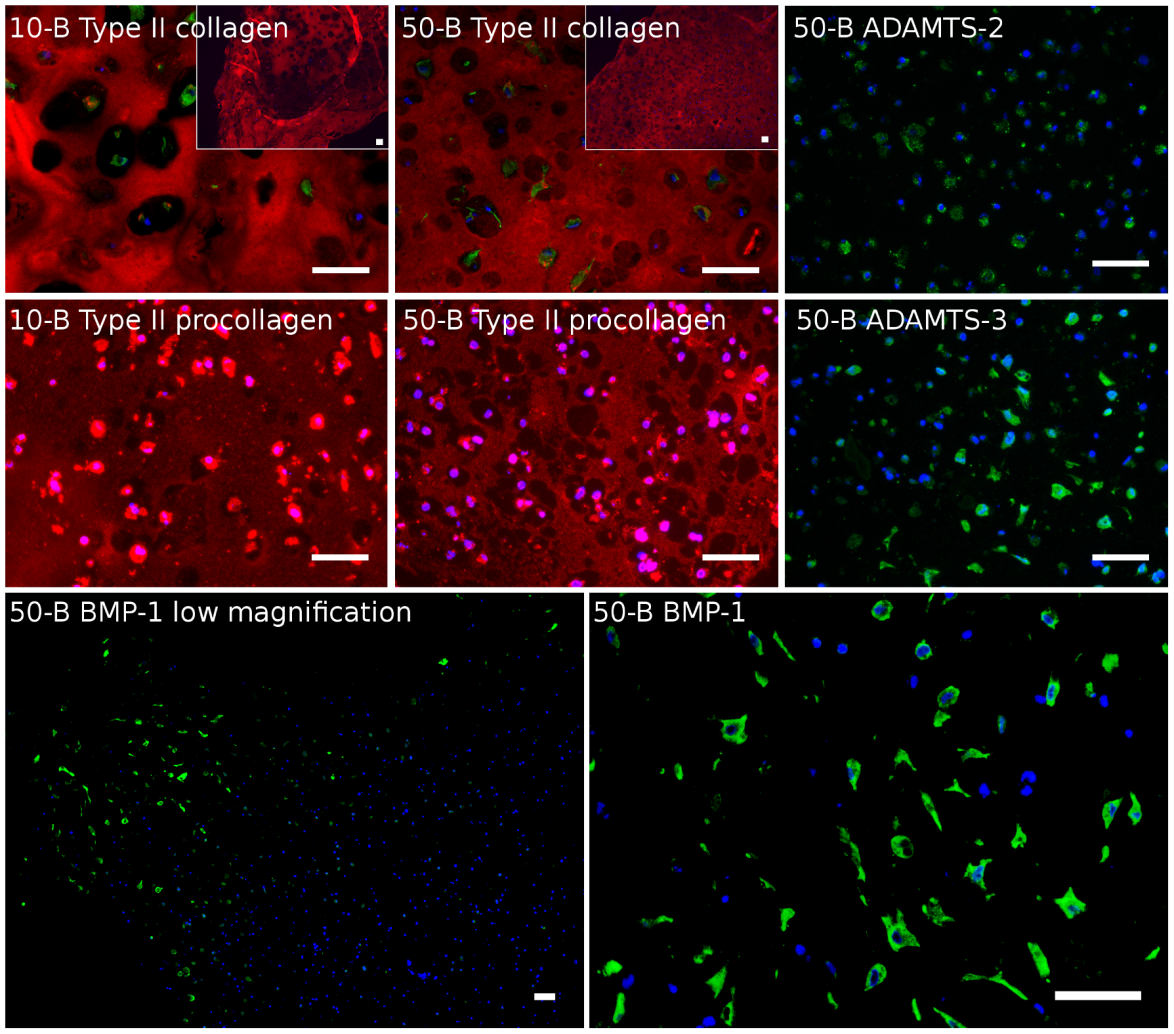
IHC staining of type II collagen (red), type II procollagen (red), ADAMTS-2 (green), ADAMTS-3 (green) and BMP-1 (green), counter-stained with DAPI (blue). In type II collagen images, pan cadherin was also stained (green). Original images of type II procollagen were captured by optimizing for signal in the ECM (*I_0_*) and the shown images were further enhanced according to 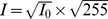. Insets show low magnification images of the respective samples. Scale bars are 50 µm.

Fluorescence IHC on samples cultured for six weeks confirmed that type II collagen (Figure 2) and aggrecan (Figure S3) were indeed secreted by the cells. At the level of whole sections (insets), the type II collagen staining was relatively homogeneous, but with some regions showing reduced staining. Those regions could typically be found in central regions of the gels. In the positive regions, type II collagen appeared to fill out the intercellular space, which indicates that the transport properties were sufficiently good for continuous structures to be formed through the alginate hydrogel matrix, even at the lower cell densities. For many of the cells, there was no staining for type II collagen in the pericellular region. This can clearly be seen in Figure 2 for sample 10-B, while for the highest cell seeding density, those regions were smaller and less pronounced. A more complete set of samples stained for type II collagen and aggrecan can be seen in Figures S2 and S3, respectively.

Selected samples stained for type VI collagen are shown in Figure S4, indicating that the pericellular region of some, but not all, of the cells contains type VI collagen.

### SHG Microscopy

**Figure 3:**
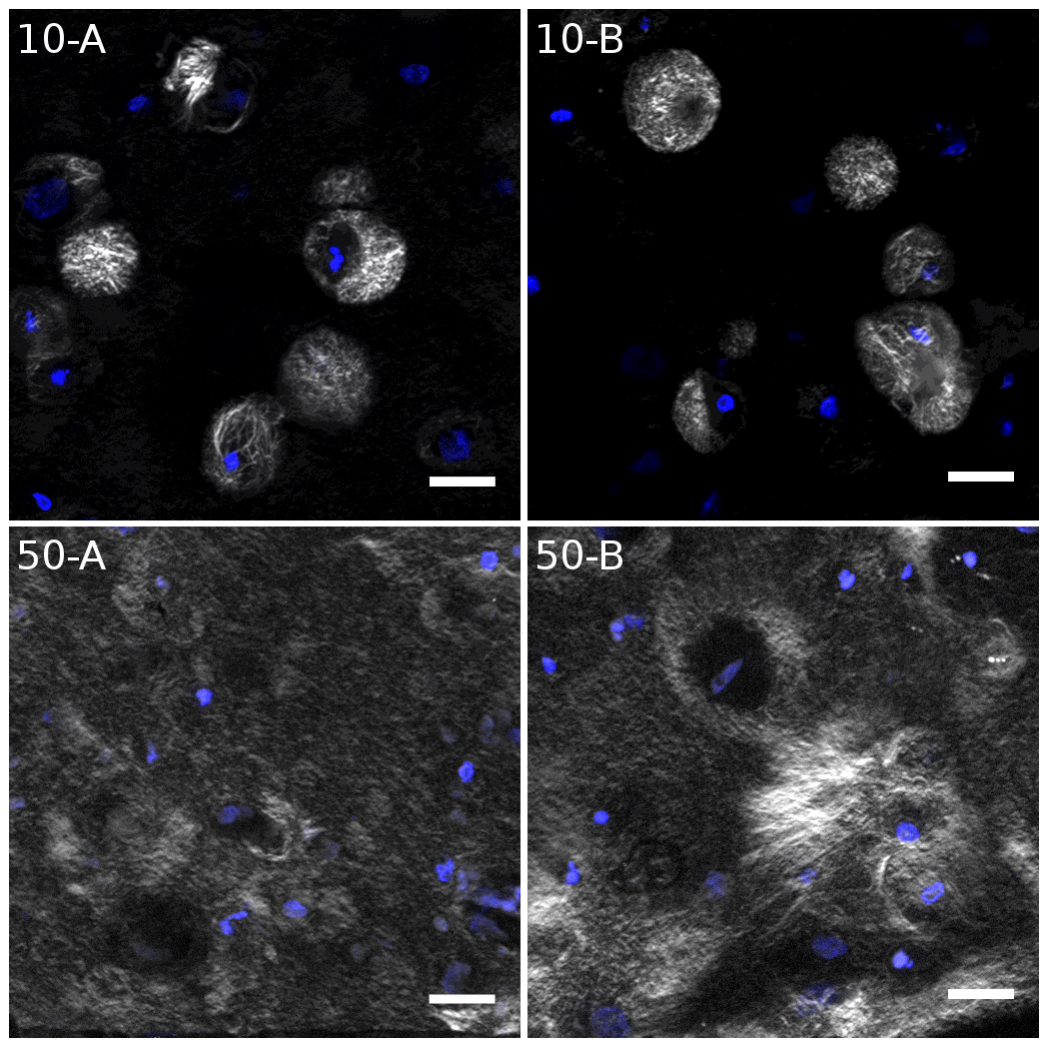
SHG microscopy of selected samples identifying fibrillar collagens (white) counter stained with DAPI (blue). 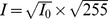
 is shown to give weight to the darker end of the detector, as described in Materials and Methods. Scale bars are 20 µm.

The SHG signal originates from non-centrosymmetric molecules and molecular assemblies, and can therefore be used to study the degree of collagen fibrillation and production of ordered matrix by the cells. Some representative micrographs acquired after six weeks of chondrogenic differentiation are shown in Figure 3, where the SHG signal is shown as white (false color) and cell nuclei have been counter stained with DAPI (blue). Samples 10-A and 10-B displayed strong pericellular SHG signal, indicating that the secreted collagens formed fibrils only in volumes up to about 30 µm in diameter. Around most of the cells showing positive SHG signal, this region showed an abrupt transition from presence to absence of fibrillar collagens. The SHG data contradicted the IHC observations described above, where type II collagen was apparently distributed well through the alginate matrix. There were no observable differences in SHG signal distribution with respect to calcium concentration in the medium. However, the hydrogels seeded with 50×10^6^ MSC ml^−1^ displayed fundamentally different distributions in SHG signal compared to the lower seeding density. It was evident that the higher seeding density could produce fibrillar collagen structures interacting over much larger distances, which may be due to higher accumulation of precursor molecules (procollagens and endopeptidases) in the extracellular space.

### TEM

**Figure 4:**
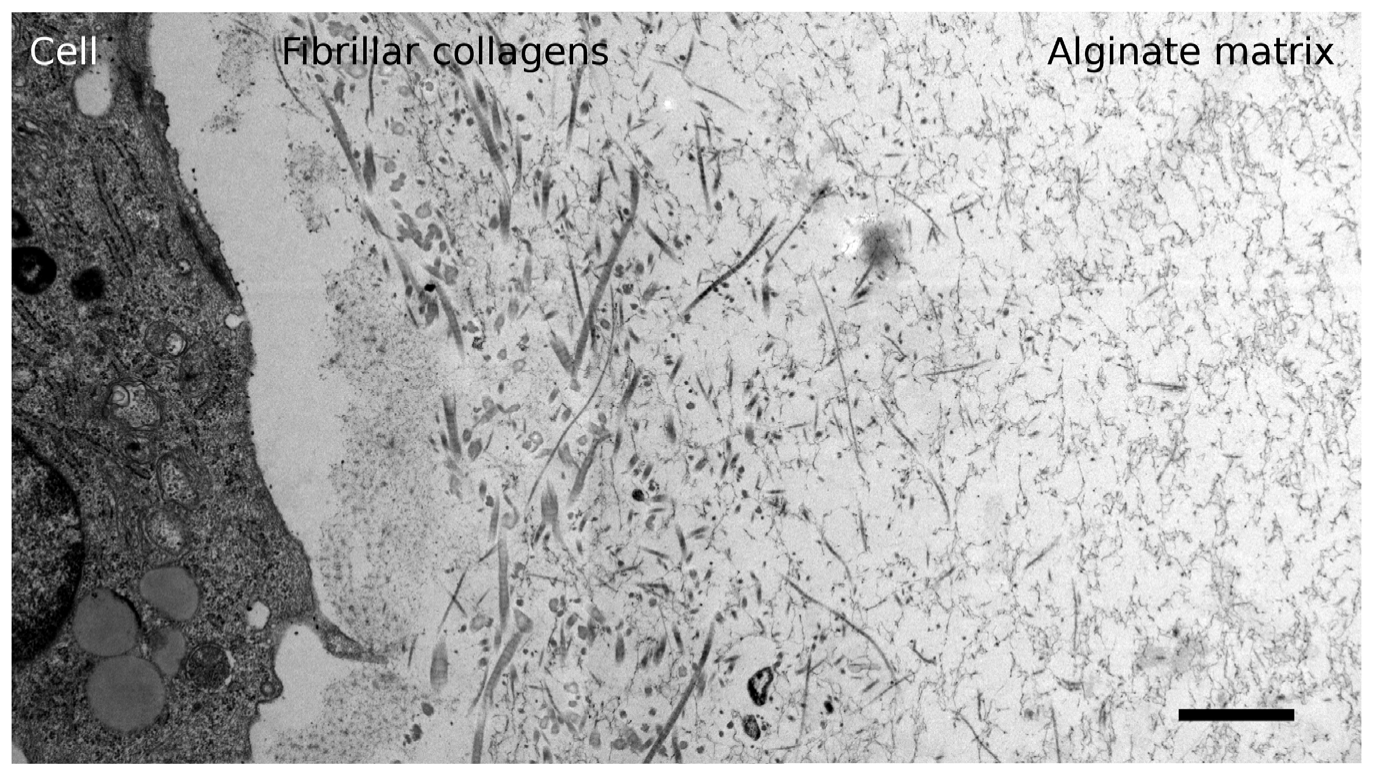
TEM micrograph (combined from two tiles) of sample 10-B after three weeks of chondrogenic differentiation, showing distinct zones of matrix radially out from the cell surface. Scale bar is 1 µm.

TEM micrographs acquired of sample 10-B after three weeks of chondrogenic differentiation (Figure 4) confirmed that the cells were forming a pericellular matrix of fibrillar collagens when seeded at 10×10^6^ ml^−1^. Further away from the cell surfaces, only a randomly oriented matrix which was most likely alginate was seen.

### FIB/SEM Tomography

**Figure 5:**
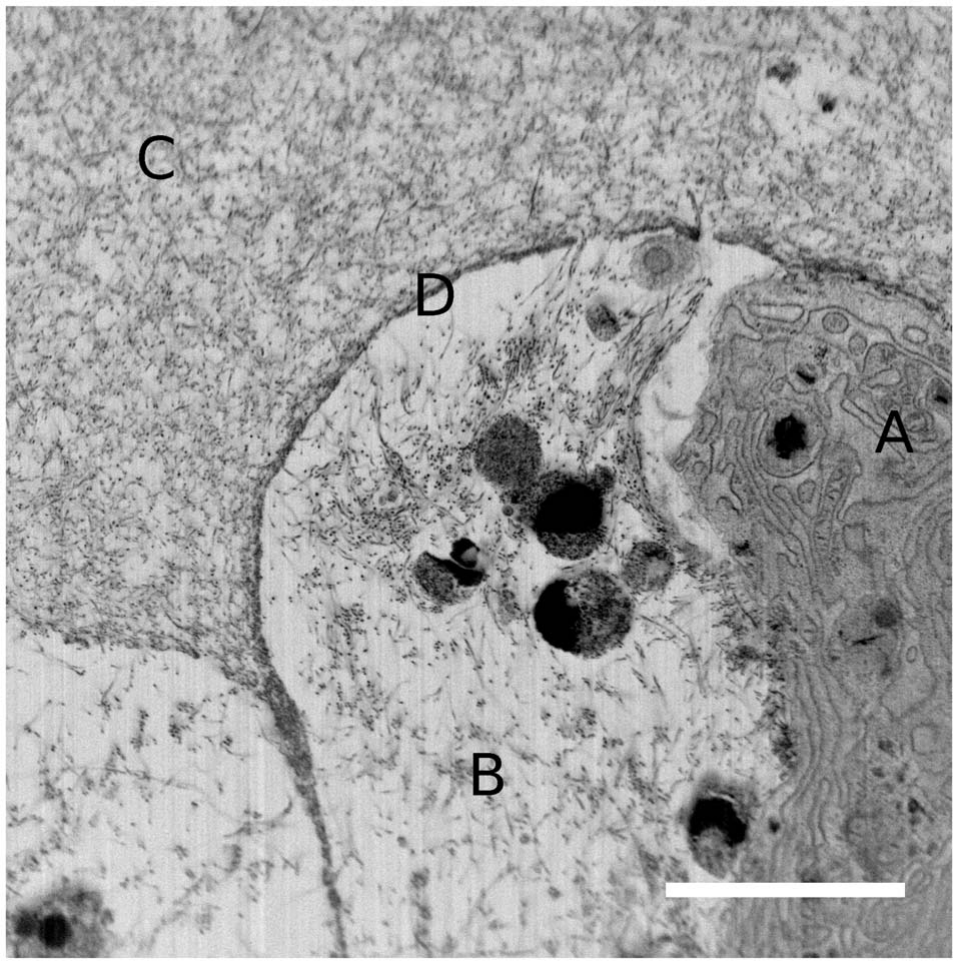
SEM micrograph (inverted colors) captured of the FIB milled sample face of sample 50-B. The four labeled zones in the micrograph are A: Cell; B: Pericellular matrix; C: ECM and; D: Boundary between pericellular matrix and ECM. Scale bar is 5 µm.

To further describe the distribution and dimensions of fibrillar collagens in the zones close to and far away from the cell surface, sample 50-B was characterized by FIB/SEM tomography after six weeks of chondrogenic differentiation. Using this method, the ultrastructure of the fibrillar collagens, inter-fibrillar interactions and the distribution through the matrix could be visualized. A volume of 20×20×6 µm^3^ was investigated, where a cell, its pericellular matrix and some distance into the ECM were included. Figure 5 shows an SEM micrograph captured of the FIB milled sample face, taken from the slice-and-view data collection. The micrograph clearly shows a cell (labeled A) with internal organelles, such as endoplasmatic reticulum and ribosomes. Outside the cell, the micrograph reveals two distinct zones; one close to the cell (B) and one extending further into the territorial/interterritorial matrix (C). The saturated black structures in zone B may be apoptotic bodies from another cell. Close examination of the matrix in area C revealed a dense network of collagen fibrils. Thicker and more organized collagen fibrils were also present in the area close to the cell surface (B). Those two zones appeared to be separated by a boundary (D), as mentioned above for the SHG microscopy, which also appears to be composed of fibrillar collagen, possibly as a result of ECM proteins being pushed against the alginate hydrogel matrix. This may explain the sharp edge of SHG signal seen in the low density cultures, where fibrillar structures were formed only in the pericellular region, before they are pushed out against the alginate boundary. For the high density cultures, however, the FIB/SEM data clearly shows that fibrillar collagens are present on both sides of this boundary.

**Figure 6:**
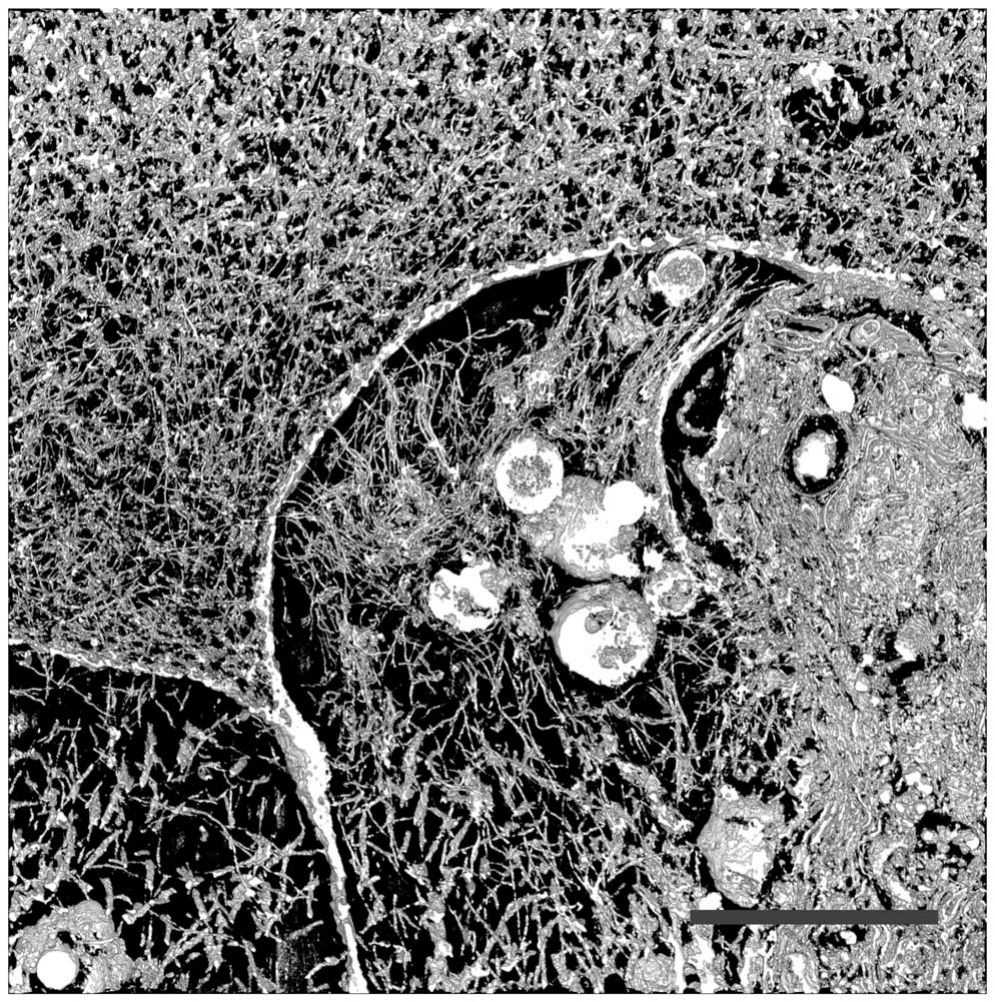
3D reconstruction based on 30 consecutive SEM micrographs, including the whole field-of-view from Figure 5, but not the full 6 µm thickness, which was done to illustrate collagen fibrils in clear detail without over-crowding of the reconstruction. An animation through the whole volume was made from snapshots of a 30 slice thick 3D model sliding through the whole volume to better visualize the full extent of all the collected data (Animation S2). Scale bar is 5 µm.

The data set obtained from slice-and-view experiments contained about 300 individual slices (SEM micrographs) collected with a separation of 20 nm, and 200 of these were used for further processing. The contrasted structures can be traced through the set of sequential images, as seen as an animation in Animation S1. The animation clearly shows that the structures were mostly relatively stiff and fibrillar extending several µm in length. The fibrils were approximately 40–50 nm in diameter. These observations suggest that the imaged structures are fibrillar collagens, and very likely type II collagen. 3D models were made from the whole field-of-view, using the 3Dviewer plugin in ImageJ, to better visualize the 3D appearance of the collagen fibrils. Figure 6 shows such a 3D model made from 30 consecutive slices. Using a limited number of slices, it was easy to resolve separate structures, although the full length of the fibrils cannot be seen. With increasing number of slices used to make the model, the fibril lengths became more evident, but the model would eventually be too crowded with structures to provide useful information (data not shown). To visualize the full set of images while retaining useful information about fibril length and network structure, an approach was developed using a volume of 30 slices sliding through the whole volume of 200 slices. The resulting animation can be viewed in Animation S2. Interestingly, the collagen fibrils can be seen both close to the cell surface and further out into the matrix. This observation supports the results from SHG microscopy, indicating that there were indeed fibrillar collagens forming further away from the cell surface when MSC were seeded at 50×10^6^ ml^−1^. Thus, there must have been collagen fibril precursor molecules transported away from the cell surface into the ECM before fibril formation was triggered, as these fibrillar structures are much too large to diffuse through the alginate matrix.

### Collagen Processing and Assembly

High cell seeding densities were required for the formation of a continuous intercellular network of fibrillar collagens. However, fluorescence IHC showed sufficiently good distribution of type II collagen even at low seeding density. To investigate the assembly of collagen fibrils further, fluorescence IHC was used to visualize the presence and distribution of type II procollagen (N-terminal region), ADAMTS-2, ADAMTS-3 and BMP-1.

Figure 2 shows micrographs of selected samples taken after three weeks of chondrogenic differentiation, where type II procollagen (N-terminal propeptide) is shown in red counterstained with DAPI (blue). The main signal from type II procollagen had a cellular localization, however, further image optimization for the extracellular regions revealed an extracellular staining pattern with similarities to those found for type II collagen (see Figure 2). IHC staining of type II procollagen, where image acquisition was optimized for the cellular localization, can be seen for a wider selection of samples in Figure S5. A negative control omitting primary antibody, which was imaged and processed the same way as the images in Figure 2 is shown in Figure S6.

Two of the enzymes relevant for N-terminal type II procollagen cleavage, ADAMTS-2 and ADAMTS-3, were probed in all the samples. Figure 2 shows selected micrographs, where the green signal is from the enzymes and the blue counterstain is from DAPI. Both enzymes were mainly found intracellularly or in close association with the cell membrane. A fraction of the cells was negative for ADAMTS-2 and ADAMTS-3, which is in agreement with the varying degree of SHG signal between cells, as described above. Images of a wider selection of samples along with isotype controls can be seen in Figures S7 and S8.

Further, the enzyme cleaving the C-terminal propeptide of type II procollagen, BMP-1, was probed for and a representative micrograph from sample 50-B is shown in Figure 2. The BMP-1 stained samples showed intracellular signal, but only a fraction of the cells stained positive. There were large areas of the stained sections that were negative for BMP-1. The active form of BMP-1 has been shown to have a size of 88 kDa [24], which results in limited transport properties in alginate hydrogels. Thus, the vast majority of cells are required to produce BMP-1 in order to efficiently process the C-terminal propeptide of all available procollagen in the alginate hydrogel. Images of a wider selection of samples along with isotype controls are shown in Figures S9 and S10.

## Discussion

The aim of this study was to investigate the collagen structures that form as MSC are immobilized in hydrogels and subjected to chondrogenic differentiation, and how these structures can potentially be influenced by cell seeding density and hydrogel dissolution kinetics. First, the chondrogenic differentiation was confirmed at the mRNA and protein levels, as gene expression and secretion of type II collagen and aggrecan were seen to be upregulated. Next, the distribution of type II collagen, as seen by fluorescence IHC, was found to be relatively homogeneous in all the samples probed in this study. However, other studies where MSCs have been immobilized in alginate for chondrogenic differentiation have shown much stronger pericellular localization of type II collagen [25], [26], even though the same antibody as in the present study was used. Both those studies immobilized 5×10^6^ MSC ml^−1^ and immersed the hydrogels in 50 mM SrCl_2_ before initiating the chondrogenic differentiation. The lower cell density compared to the present study may result in sufficiently long distances between cells to yield an inhomogeneous distribution of the secreted type II collagen. Also, when alginate is cross-linked by a combination of Ca^2+^ and Sr^2+^ ions, a higher cross-linking density is obtained than for Ca^2+^ alone, because shorter blocks of guluronic acid are sufficient to form stable cross-links [27]. Such higher cross-linking densities will result in slower transport of macromolecules through the hydrogel.

Transport of secreted ECM proteins through the hydrogel matrix for assembly of a functional and continuous matrix is a fundamental issue in tissue engineering when a biomaterial matrix is used to immobilize ECM-producing cells. For chondrogenic differentiation and formation of a functional cartilage matrix, adequate ECM protein transport properties must be combined with properties of the immobilization matrix that allow for a rounded cell morphology, which is a characteristic of the chondrocyte phenotype. For poly(ethylene glycol) hydrogels, it has been shown that increasing polymer densities efficiently limit the transport of ECM molecules through the gel, resulting in strong pericellular localizations, even of the relatively small glycosaminoglycans [5]. Another study, using alginate hydrogels as immobilization matrix [28], argued differently on the issue of ECM molecule transport. They found procollagens escaping the hydrogels to the culture medium, and argued that both procollagen and BMP-1, being negatively charged, can diffuse through the gel, which is also negatively charged. If procollagen molecules diffuse away from the cell surface prior to being processed by endopeptidases, collagen fibril assembly is not feasible. Following these arguments, a pericellular matrix is allowed to be formed, because the endopeptidase concentration is high enough to process sufficient amounts of procollagen close to the cell surface, but further away from the cell surface procollagen molecules will remain in solution. In agreement with that study, we showed by fluorescence IHC that type II procollagen was indeed distributed through the alginate hydrogel matrix, yielding similar staining patterns to those shown for type II collagen.

Type II collagen molecules, and, to a much greater extent, collagen fibrils, have limited diffusivity in alginate hydrogels, where the pore size is small. It has been shown by N_2_ adsorption and TEM image analysis [29], that, in 1.4% alginate hydrogels, approximately 60% of the pores have radii smaller than 10 nm. In comparison, the triple-helical collagen molecule is approximately 1.5 nm in diameter and 300 nm in length [30], indicating that even single collagen molecules will have limited transport properties in alginate hydrogels. The use of a culture medium with reduced calcium concentration was expected to improve the distribution of type II collagen, because the low calcium concentration results in slow dissolution of the alginate hydrogels, and therefore may provide more space for transportation of larger molecules. No such effect could be observed in the present study.

The N-terminal procollagen peptidases ADAMTS-2 and ADAMTS-3 showed intracellular localization by fluorescence IHC in the present study. Combined with the presence of type II procollagen in the ECM (shown with an antibody specific for a sequence in the N-terminal part of type II procollagen), there was clearly a fraction of the type II procollagen molecules that remained unprocessed and, therefore, was unable to form fibrils. The SHG microscopy showed that there was, indeed, a spatial variation in the assembly of collagen fibrils for the low seeding density samples, which was also confirmed by TEM. To support these observations, it has been shown by mathematical modeling [31] that a minimum cell seeding density of about 23×10^6^ cells ml^−1^ is required for a continuous ECM to be formed, given that the secreted proteins have a certain diffusivity before they are immobilized to become functional ECM. The combination of high cell seeding density, lower hydrogel density and tailored degradation kinetics may therefore provide a better way to engineer functional cartilage tissue.

There were only small differences in distribution of type II collagen and aggrecan between low and high cell seeding densities observed by IHC, although SHG showed clear differences. A possible reason for such observations may be the lack of specificity of the used antibodies for functional molecular structures as compared to the same molecules merely in solution. E.g. for type II collagen, it may very well be that the antibodies bind to both fibrillar collagen, collagen in solution, procollagen in solution and collagen degradation products from MMP activity. Staining of procollagen in solution is probable, as discussed above for the antibody specific to N-terminal type II procollagen. MMP activity may be supported by the lack of type II collagen staining in the pericellular space for low cell seeding densities, which may be a result of continuous degradation of ECM structures forming in these regions. The products of MMP-degraded fibrillar type II collagen are probably small enough to facilitate sufficiently good transport properties for these fragments to be distributed throughout the alginate hydrogel matrix, for subsequent detection by the type II collagen antibody. MMP activity was also supported by the upregulation of MMP-13 at the mRNA level, as shown by RT qPCR.

Further, in agreement with mathematical modeling [31], the hydrogels seeded with 50×10^6^ MSC ml^−1^ displayed a more homogeneous distribution of SHG signal compared to the pericellular localization seen for the lowest seeding density. This indicates that collagen fibrils were formed further away from the cell surface, penetrating into the ECM, resulting in a much more continuous network of fibrillar collagens. The FIB/SEM observations clearly showed that collagen fibrils with diameter of approximately 40–50 nm and length of several µm were present in the samples seeded with 50×10^6^ MSC ml^−1^, both in the pericellular region and extending into the ECM. The physical dimensions of these fibrillar structures suggest that they cannot be transported effectively through the alginate hydrogel matrix by diffusion [29], but must be assembled at the location at which they were found in the FIB/SEM data. This means, in turn, that the fibril precursor molecules (soluble type II (pro)collagen) must be transported away from the cell surface, into the alginate matrix, and to the location at which they are to be assembled into larger structures. There are several mechanisms by which such transport can be accomplished. Either, one or both of the N- and C-terminal propeptides remain bound to the collagen molecule, thereby rendering the fibril precursor soluble until the peptides are cleaved off at the location of fibril assembly. Such a mechanism requires procollagen endopeptidases to be present in the ECM after secretion from the cells. Both ADAMTS-2, ADAMTS-3 and BMP-1 were found by IHC in the samples, but the localization was intracellular. As these enzymes have extracellular functions, and because we see collagen fibrils in the ECM, we must assume that they are also secreted. However, we did not manage to demonstrate that. In addition, BMP-1 was found only in a fraction of the cells, which identifies this enzyme as a potential target for new tissue engineering strategies, possibly by exogenous addition of BMP-1 to the biomaterial. The enzymes range in size approximately between 70–130 kDa [13], thus having limited transport potential in alginate hydrogels. Supported by the SHG and FIB/SEM observations of fibrillar collagens in the ECM, it is likely that type II procollagen, ADAMTS-2/3 and BMP-1 are secreted and, although limited, diffuse to a certain extent through the alginate matrix. Increasing the cell seeding density five-fold (from 10 to 50×10^6^ MSC ml^−1^) may therefore yield sufficiently high concentrations and satisfactory distribution of these proteins, in order to facilitate the assembly of a functional fibrillar type II collagen matrix. Another possible mechanism may be stabilization of already processed type II collagen molecules in solution by small proteoglycans, which then accommodate transport into the ECM before fibril assembly. It is known that collagen has a large number of binding partners in vivo, and these may play important roles in facilitating the transport to the site of fibril assembly. Incorporation of small proteoglycans in the hydrogel may be another option to promote and modulate transport and assembly of collagen fibrils.

3D modeling based on FIB/SEM sequential imaging has recently emerged as a promising technique to study tissues [22], [23], cells, cell-substrate [32] and cell-tissue [33] interactions, where it is important to map the 3D architecture of interactions and structures over significant distances. To our knowledge, articular cartilage has been imaged using FIB/SEM instrumentation only after paraffin embedding, with no staining and not with the aim of producing serial/3D image data sets [33]. To gain knowledge about the 3D structure of chondrocytes and their peri- and extracellular matrices, with resolution in the nanometer range, proper fixation, staining and embedding are required. A thorough FIB/SEM study investigating the structures in the different layers of articular cartilage should be carried out in the future, and would be an important reference point of fibrillar collagen ultrastructure in relation to the chondrocyte and its peri-/extracellular environment. The FIB/SEM data presented in this work, represents a new and promising way to study ECM in relation to ECM-producing cells in tissue engineering, and allows for a better understanding of the structure of collagen fibril networks spanning over significant distances in 3D.

The focus of the presented work was to investigate matrix formation in tissue engineered cartilage, while mechanical properties of the forming material were not characterized. We expect that the changes in spatial arrangement of collagen fibrils, as observed by SHG microscopy and FIB/SEM tomography, are indicative of a positive change in mechanical properties for high cell seeding densities compared to the pericellular collagen fibril localization for low seeding density. It may also be possible to improve the distribution of collagen fibrils by means of mechanical stimulation, which would increase the flow of liquid in the hydrogel and thus improve the mass transport of ECM precursor molecules. A thorough study characterizing the viscoelastic properties of tissue engineered cartilage with different collagen fibril organizations acquired by means of cell seeding density and mechanical stimulation should be undertaken in the future.

## Conclusion

MSC undergoing chondrogenic differentiation in alginate hydrogels were shown to secrete relevant proteins for articular cartilage tissue formation. Particularly, type II collagen was found by fluorescence IHC to distribute relatively homogeneously through the alginate matrix. However, at low cell seeding densities, collagen fibrils were only formed in the pericellular region, as seen by SHG microscopy and TEM. The discrepancy between fluorescence IHC and SHG/TEM may be caused by IHC staining of soluble type II procollagen and type II collagen degradation products, the latter being a result of MMP activity. Increased cell seeding densities significantly improved the distribution of SHG signal from fibrillar collagens, but reduced calcium concentration in the medium to allow for dissolution of the alginate hydrogel did not further improve these results. FIB/SEM tomography on samples with high cell seeding density resolved individual collagen fibrils and their distribution in the regions close to a cell and extending into the ECM. Those results clearly showed that the dimensions of the formed fibrils were too large to be transported through the alginate hydrogel matrix, and the fibrils found in the ECM must therefore have been formed at the location where they were seen. We believe that this is possible by (1) secretion of type II procollagen with one or both of the N- and C-terminal propeptides unprocessed, followed by (2) secretion of BMP-1 and/or ADAMTS-2/3, (3) transport of procollagen, ADAMTS-2/3 and/or BMP-1 into the ECM, where final procollagen processing occurs and (4) collagen fibrils self-assemble and are thus immobilized. This sequence of events requires the concentrations of both procollagen, ADAMTS-2/3 and/or BMP-1 to be sufficiently high on site for final processing to occur, and, therefore, can only happen when cell seeding density is sufficiently high. It should be noted that there are other molecules, such as small proteoglycans, which may play important roles in collagen fibril assembly as well.

## Supporting Information

Figure S1 **FIB (a) and SEM (b) micrographs of the prepared sample volume before FIB/SEM characterization was started.** The FIB micrograph shows the top surface, while the SEM micrograph is angled 52° and, thus, shows the smooth surface were the sample has been exposed.
(TIF)

Figure S2 **IHC staining of type II collagen (red), pan cadherin (green) and nuclear stain (blue) after 6 weeks of chondrogenic differentiation.** Scale bars are 50 µm.
(TIF)

Figure S3 **IHC staining of aggrecan (red), pan cadherin (green) and nuclear stain (blue) after 6 weeks of chondrogenic differentiation.** Scale bars are 50 µm.
(TIF)

Figure S4 **IHC staining of type VI collagen (red), pan cadherin (green) and nuclear stain (blue) of selected samples.** Type VI collagen has formed a pericellular matrix around many of the cells. Scale bars are 50 µm.
(TIF)

Figure S5 **IHC staining of type II procollagen (red) and nuclear stain (blue) after three and six weeks of chondrogenic differentiation in alginate.** Scale bars are 50 µm.
(TIF)

Figure S6 **Negative control for type II procollagen omitting primary antibody.** The image was captured using the same settings and the same post-processing as for the type II procollagen staining shown in Figure 2 in the article. Scale bar is 50 µm.
(TIF)

Figure S7 **IHC staining of ADAMTS-2 (green) and nuclear stain (blue) after three and six weeks of chondrogenic differentiation in alginate.** Scale bars are 50 µm.
(TIF)

Figure S8 **IHC staining of ADAMTS-3 (green) and nuclear stain (blue) after three and six weeks of chondrogenic differentiation in alginate.** Scale bars are 50 µm.
(TIF)

Figure S9 **IHC staining of BMP-1 (green) and nuclear stain (blue) after three and six weeks of chondrogenic differentiation in alginate.** Scale bars are 50 µm.
(TIF)

Figure S10 **IHC staining of BMP-1 (green) and nuclear stain (blue) after three and six weeks of chondrogenic differentiation in alginate.** Scale bars are 200 µm.
(TIF)

Animation S1 **SEM micrographs collected by Slice-and-View, where FIB milling and SEM imaging were done sequentially through the probed sample volume.** Scale bar is 5 µm.
(AVI)

Animation S2 **3D reconstruction based on 30 consecutive SEM micrographs, including the whole field-of-view from** **Figure 5** **, but not the full 6 µm thickness, which was done to illustrate collagen fibrils in clear detail without over-crowding of the reconstruction.** An animation through the whole volume was made from snapshots of a 30 slice thick 3D model sliding through the whole volume to better visualize the full extent of all the collected data. Scale bar is 5 µm.
(AVI)
