# Correction: Biochemical and Structural Characterization of Neocartilage Formed by Mesenchymal Stem Cells in Alginate Hydrogels

**DOI:** 10.1371/journal.pone.0101096

**Published:** 2014-06-19

**Authors:** The PLOS ONE Staff

The supplementary images for Figures S6, S7, and S8 are switched. Please refer to Figure S6 for the correct Figure S7 image, refer to Figure S7 for the correct Figure S8 image, and refer to Figure S8 for the correct Figure S6 image. The Supplementary Figures in their correct order can be viewed below.

## Supporting Information

Figure S6 **Negative control for type II procollagen omitting primary antibody.** The image was captured using the same settings and the same post-processing as for the type II procollagen staining shown in Figure 2 in the article. Scale bar is 50 µm.
(TIF)

Figure S7 **IHC staining of ADAMTS-2 (green) and nuclear stain (blue) after three and six weeks of chondrogenic differentiation in alginate**. Scale bars are 50 µm.
(TIF)

Figure S8 **IHC staining of ADAMTS-3 (green) and nuclear stain (blue) after three and six weeks of chondrogenic differentiation in alginate.** Scale bars are 50 µm.
(TIF)
