# Supplementary figures and images for: Biochemical and Structural Characterization of Neocartilage Formed by Mesenchymal Stem Cells in Alginate Hydrogels
Source: PLoS One. 2014 Mar 13;9(3):e91662. doi: 10.1371/journal.pone.0091662 (PMC3953515; doi:10.1371/journal.pone.0091662)

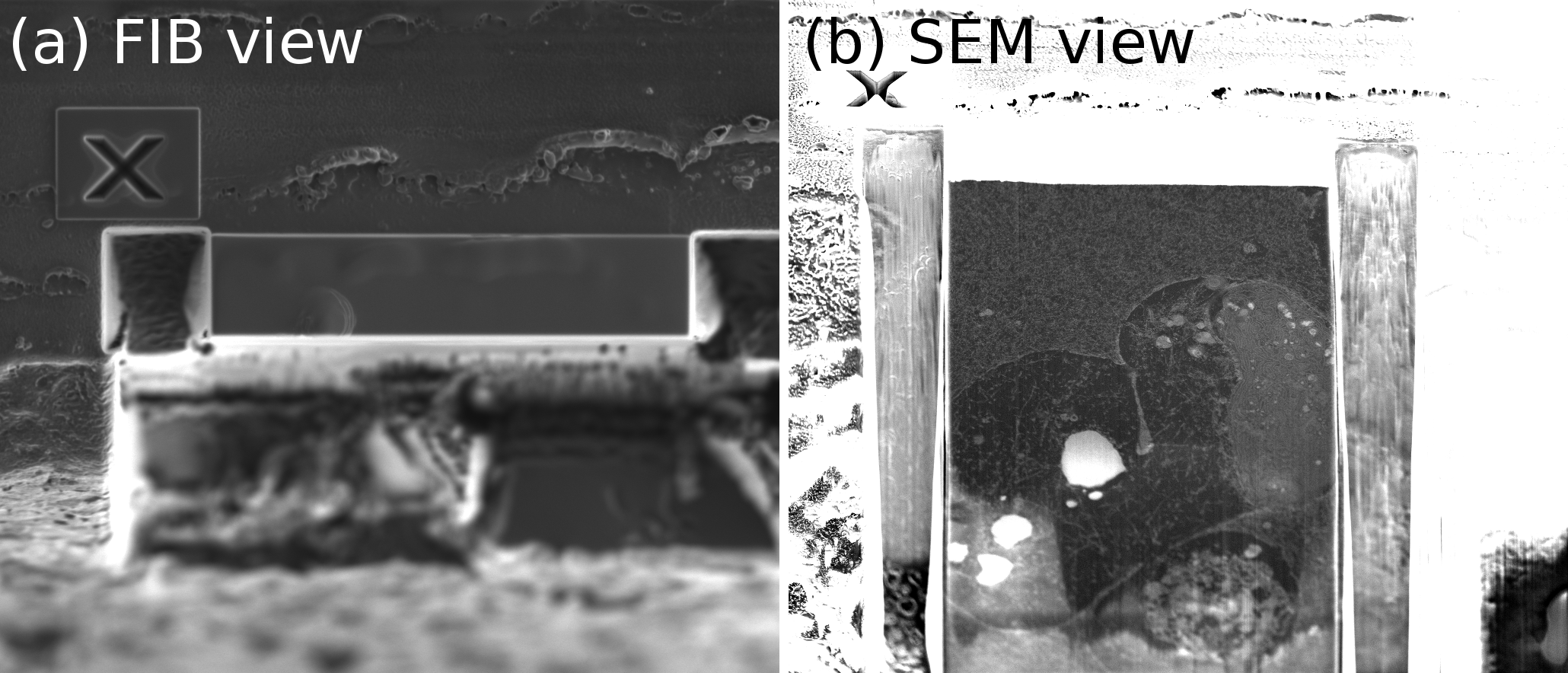

Supplement: Figure S1 — FIB (a) and SEM (b) micrographs of the prepared sample volume before FIB/SEM characterization was started. The FIB micrograph shows the top surface, while the SEM micrograph is angled 52° and, thus, shows the smooth surface were the sample has been exposed. (TIF) [file pone.0091662.s001.tif]

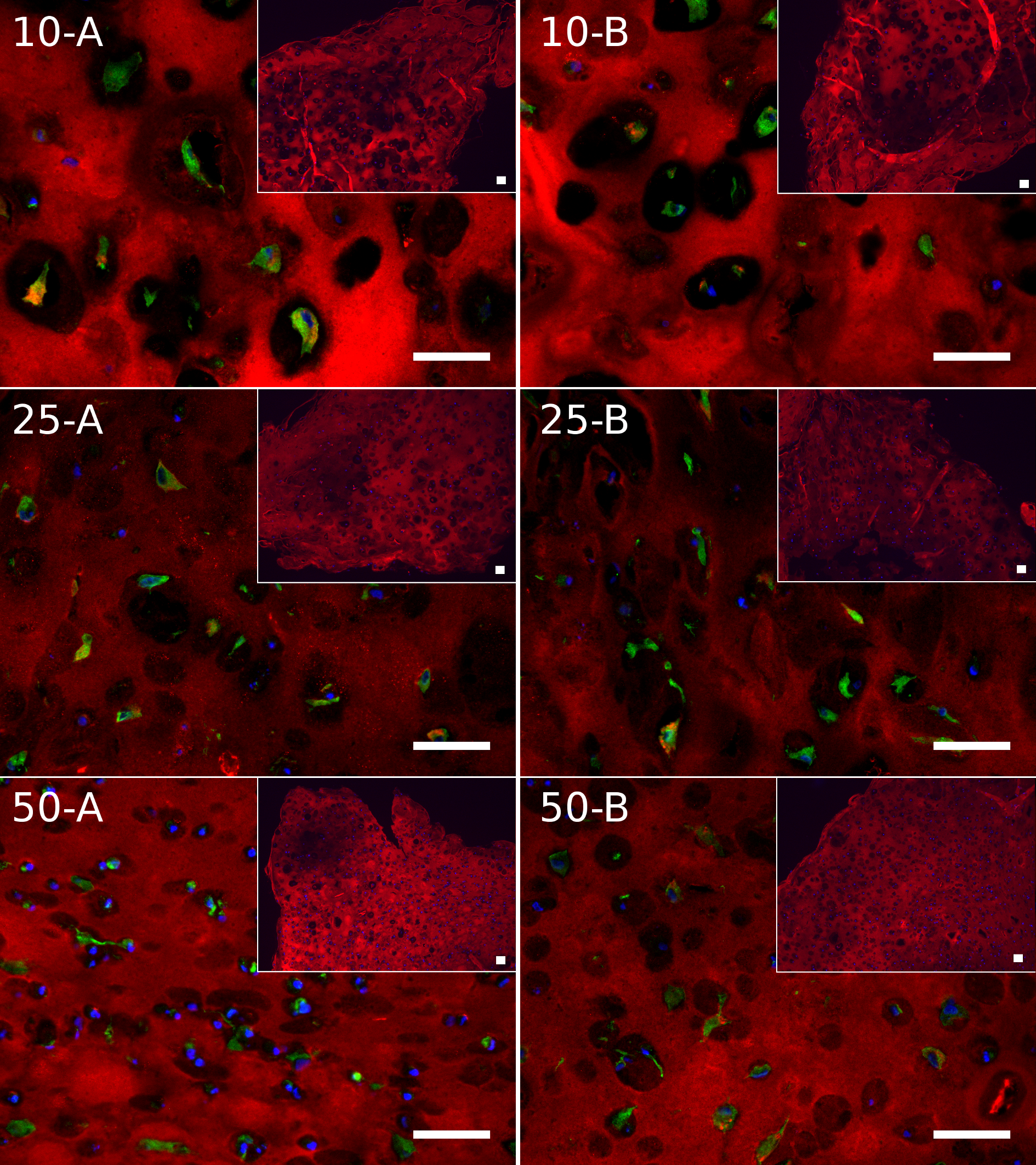

Supplement: Figure S2 — IHC staining of type II collagen (red), pan cadherin (green) and nuclear stain (blue) after 6 weeks of chondrogenic differentiation. Scale bars are 50 µm. (TIF) [file pone.0091662.s002.tif]

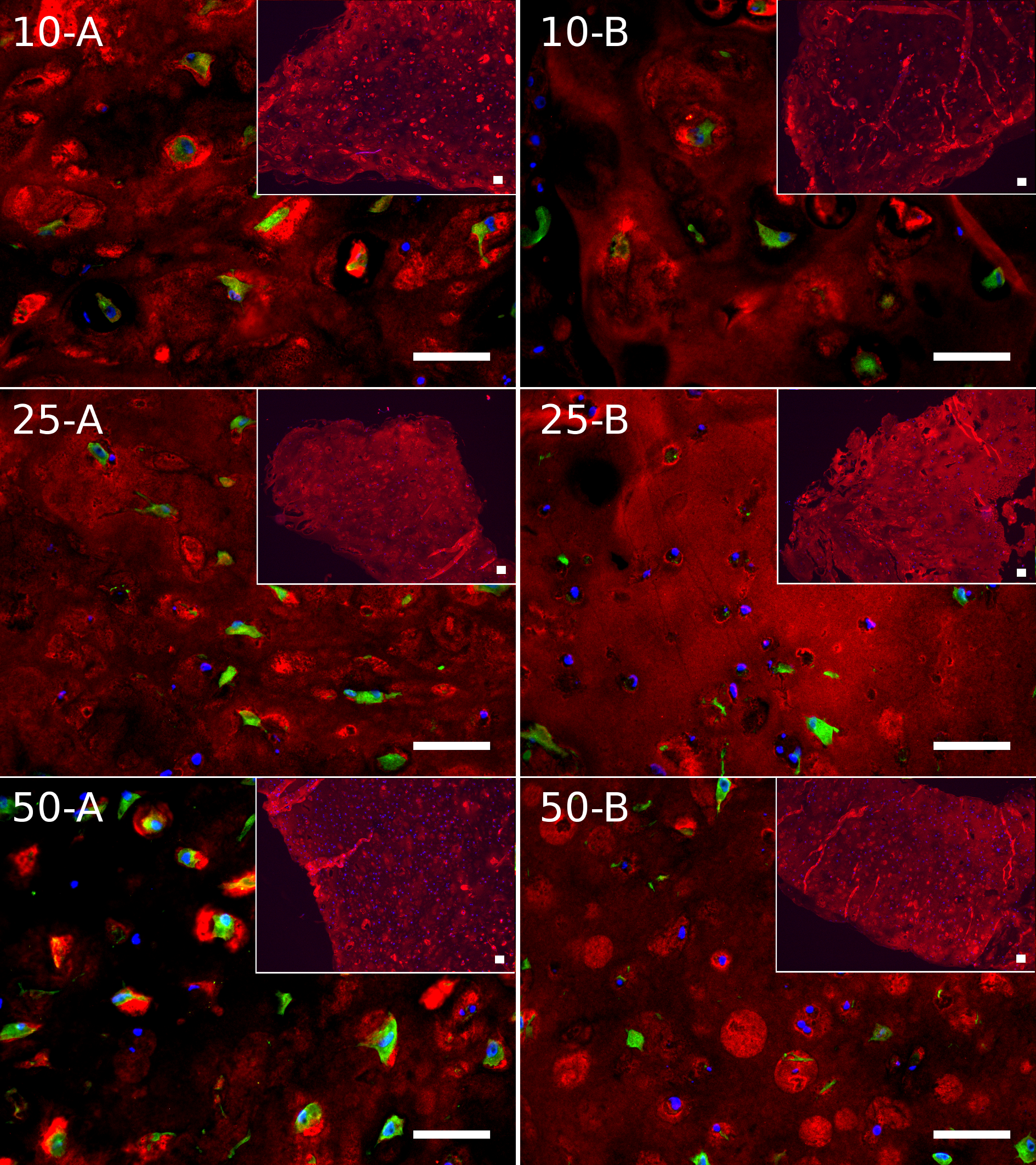

Supplement: Figure S3 — IHC staining of aggrecan (red), pan cadherin (green) and nuclear stain (blue) after 6 weeks of chondrogenic differentiation. Scale bars are 50 µm. (TIF) [file pone.0091662.s003.tif]

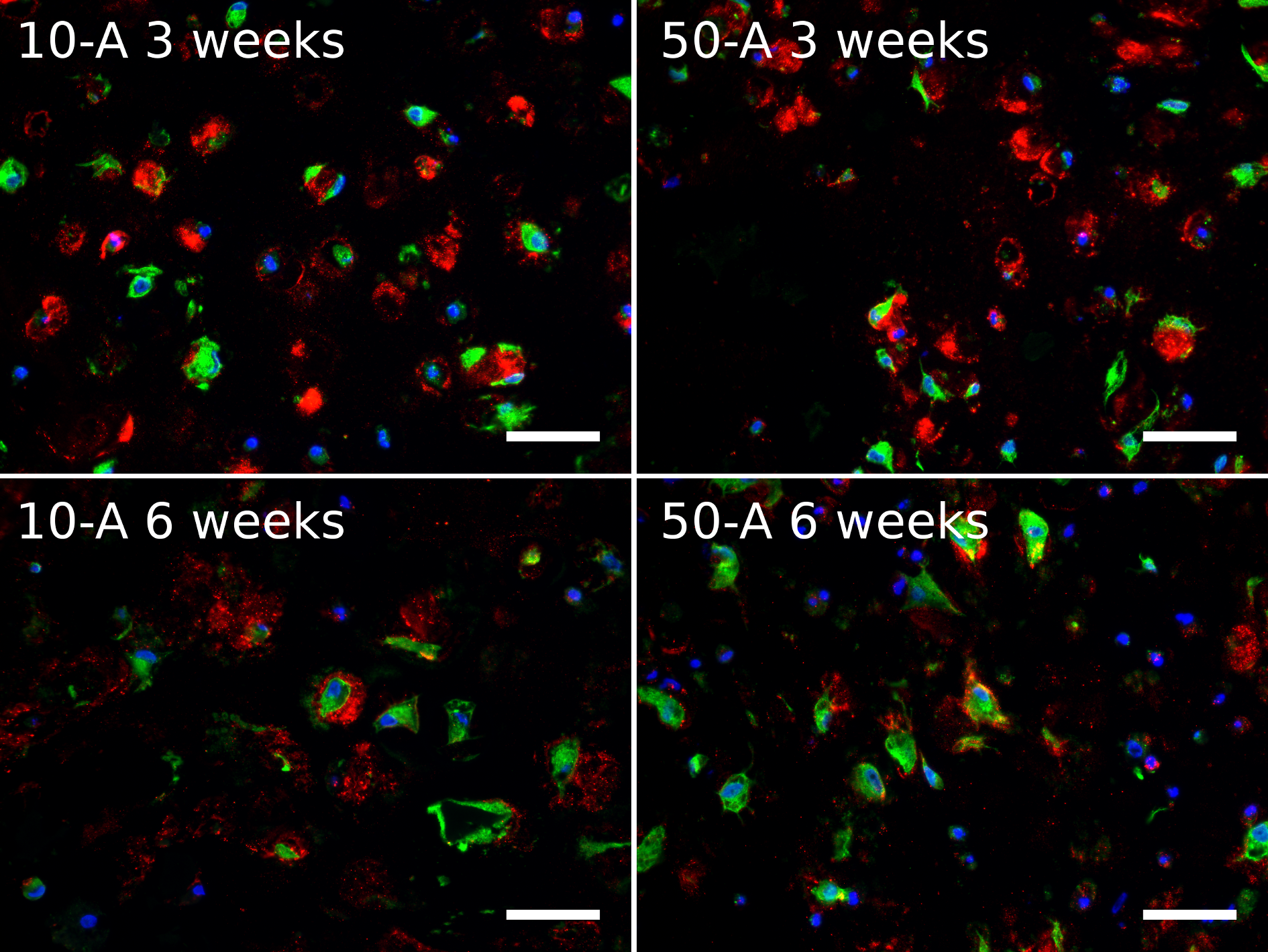

Supplement: Figure S4 — IHC staining of type VI collagen (red), pan cadherin (green) and nuclear stain (blue) of selected samples. Type VI collagen has formed a pericellular matrix around many of the cells. Scale bars are 50 µm. (TIF) [file pone.0091662.s004.tif]

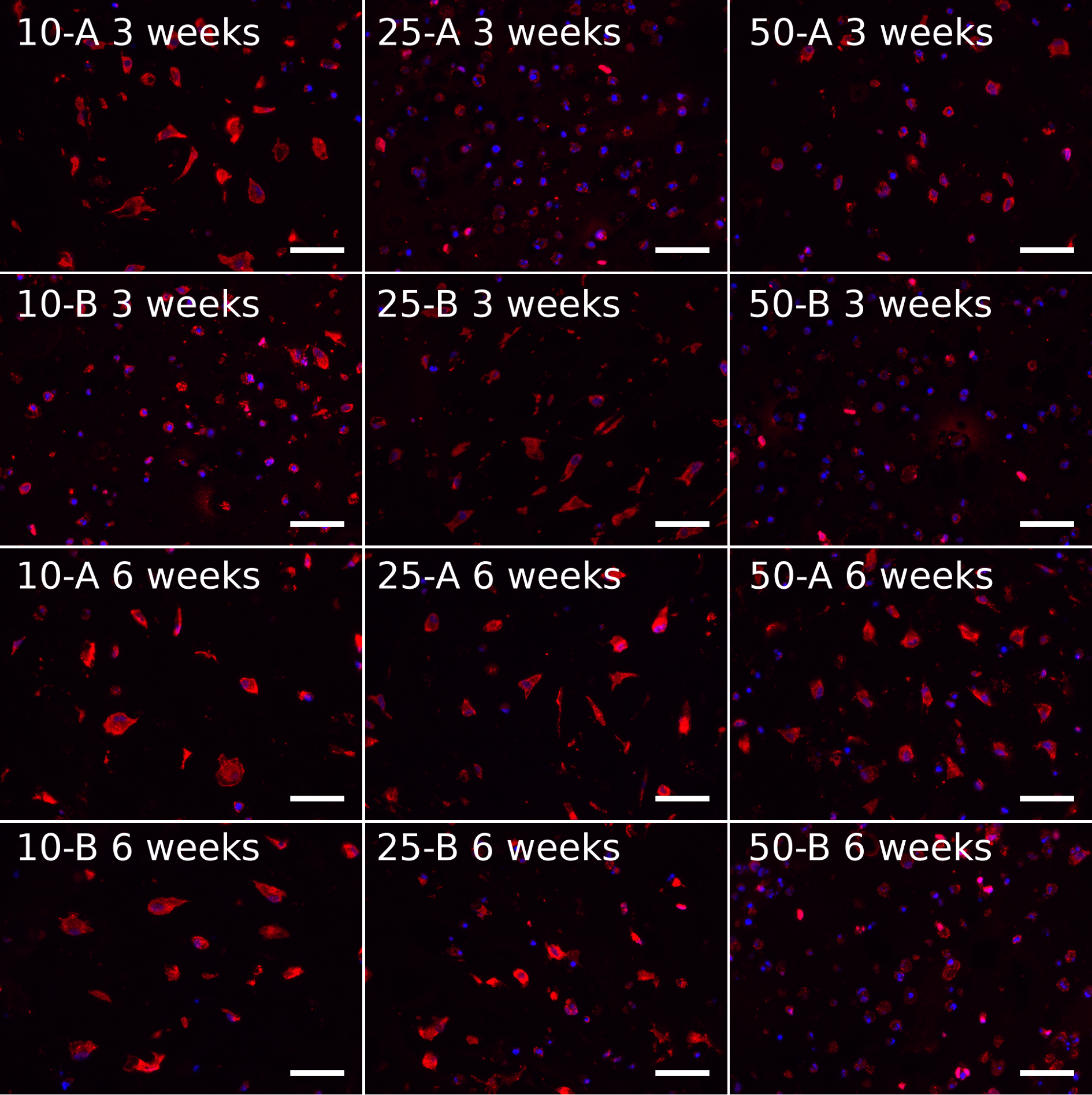

Supplement: Figure S5 — IHC staining of type II procollagen (red) and nuclear stain (blue) after three and six weeks of chondrogenic differentiation in alginate. Scale bars are 50 µm. (TIF) [file pone.0091662.s005.tif]

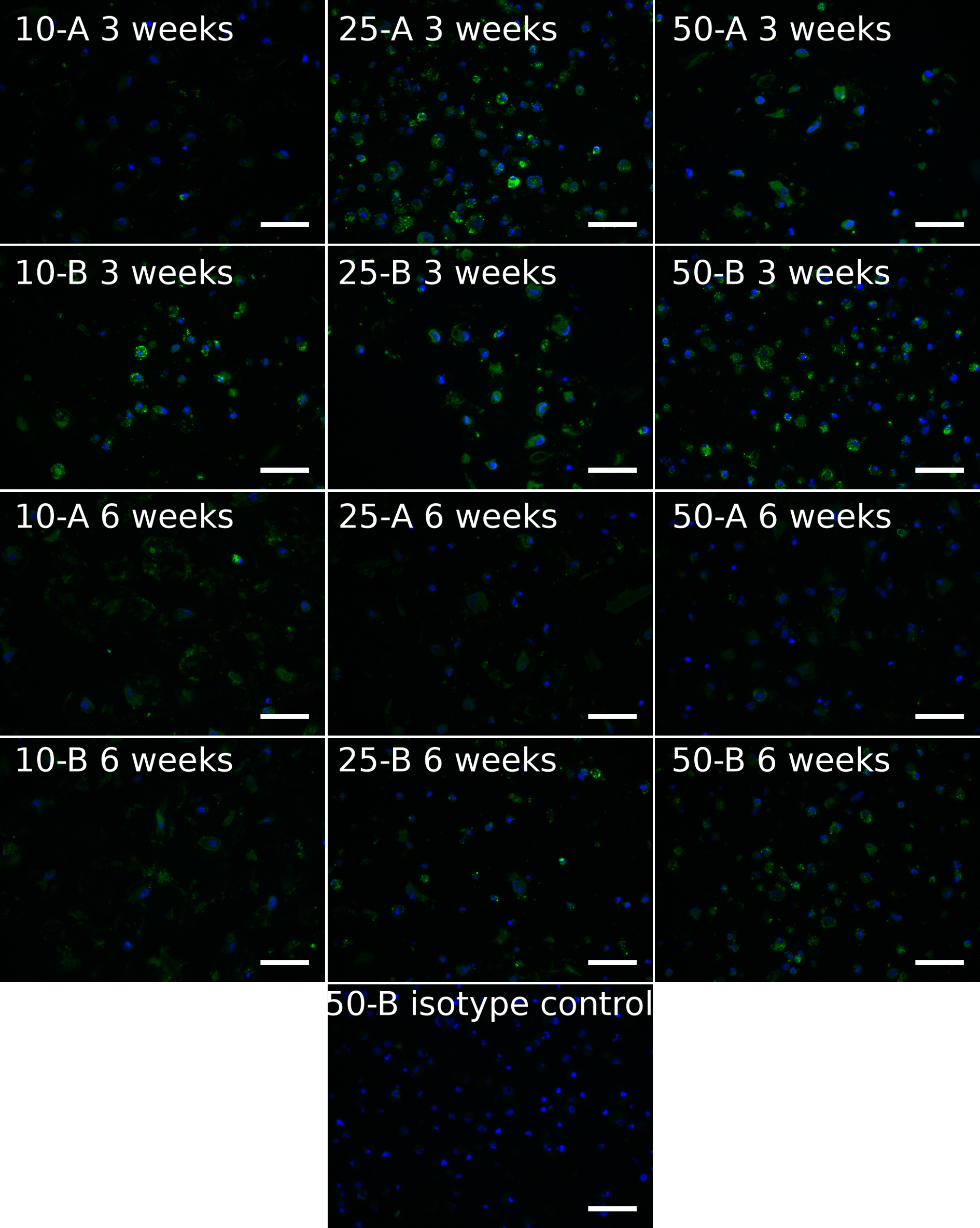

Supplement: Figure S6 — Negative control for type II procollagen omitting primary antibody. The image was captured using the same settings and the same post-processing as for the type II procollagen staining shown in Figure 2 in the article. Scale bar is 50 µm. (TIF) [file pone.0091662.s006.tif]

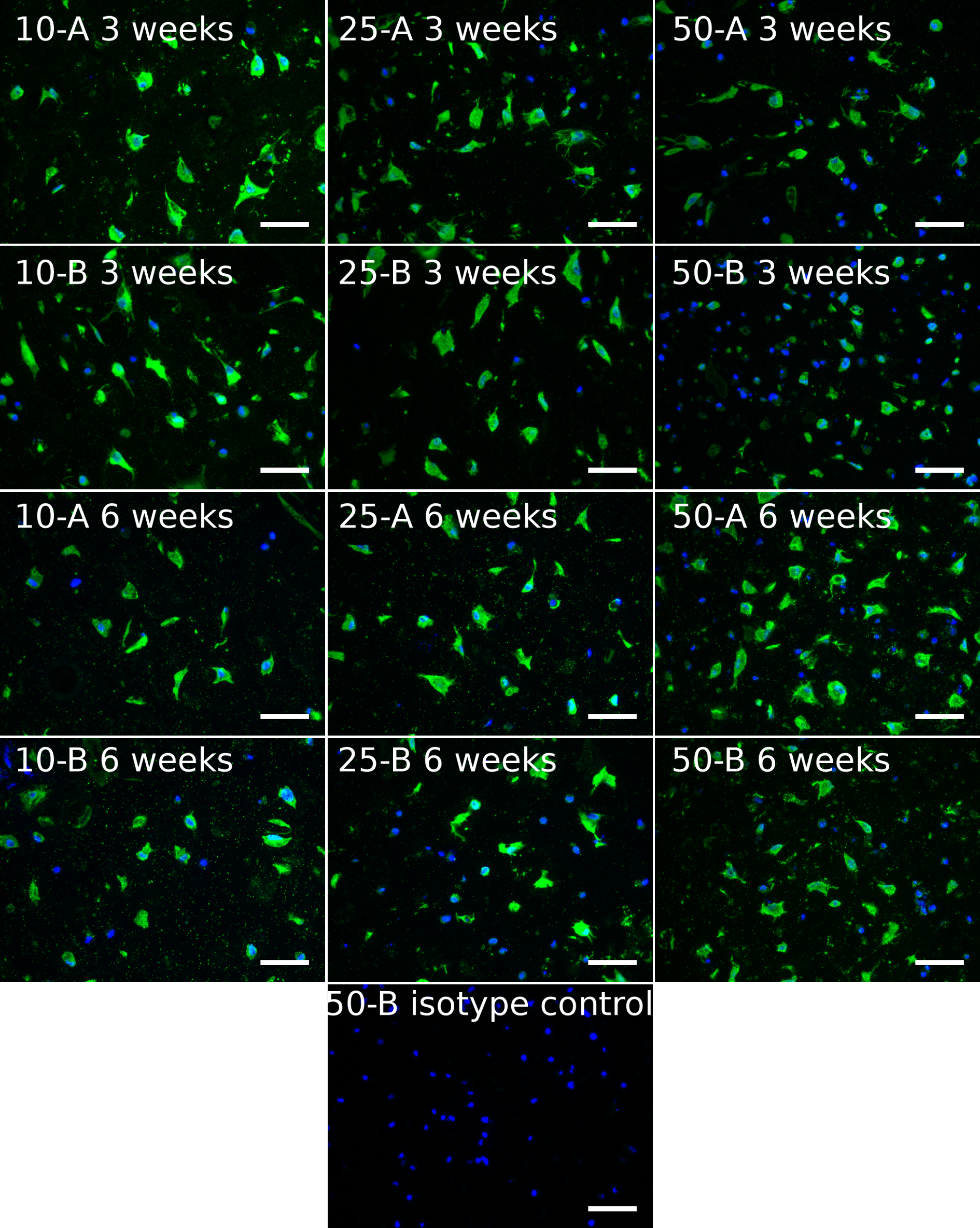

Supplement: Figure S7 — IHC staining of ADAMTS-2 (green) and nuclear stain (blue) after three and six weeks of chondrogenic differentiation in alginate. Scale bars are 50 µm. (TIF) [file pone.0091662.s007.tif]

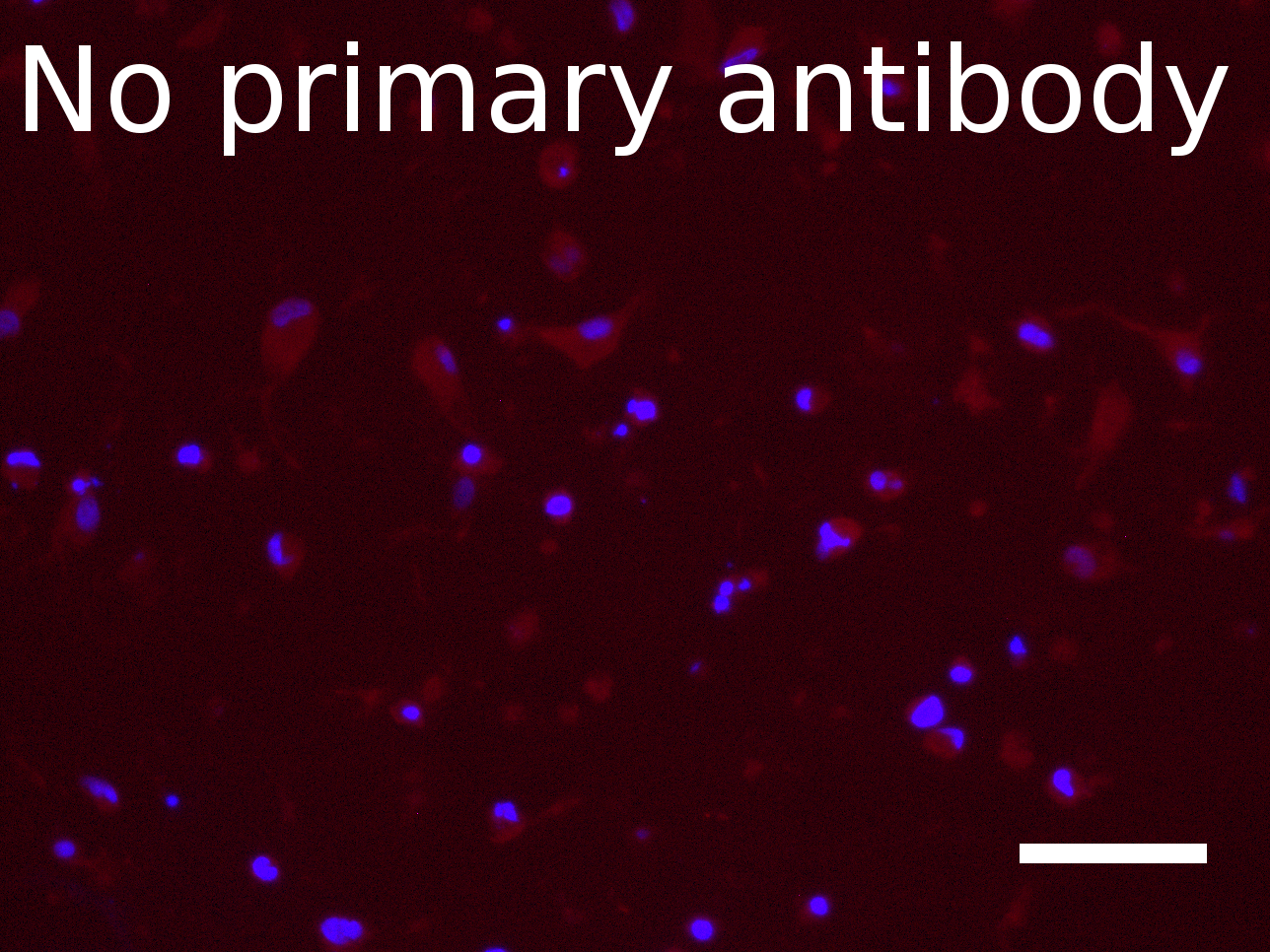

Supplement: Figure S8 — IHC staining of ADAMTS-3 (green) and nuclear stain (blue) after three and six weeks of chondrogenic differentiation in alginate. Scale bars are 50 µm. (TIF) [file pone.0091662.s008.tif]

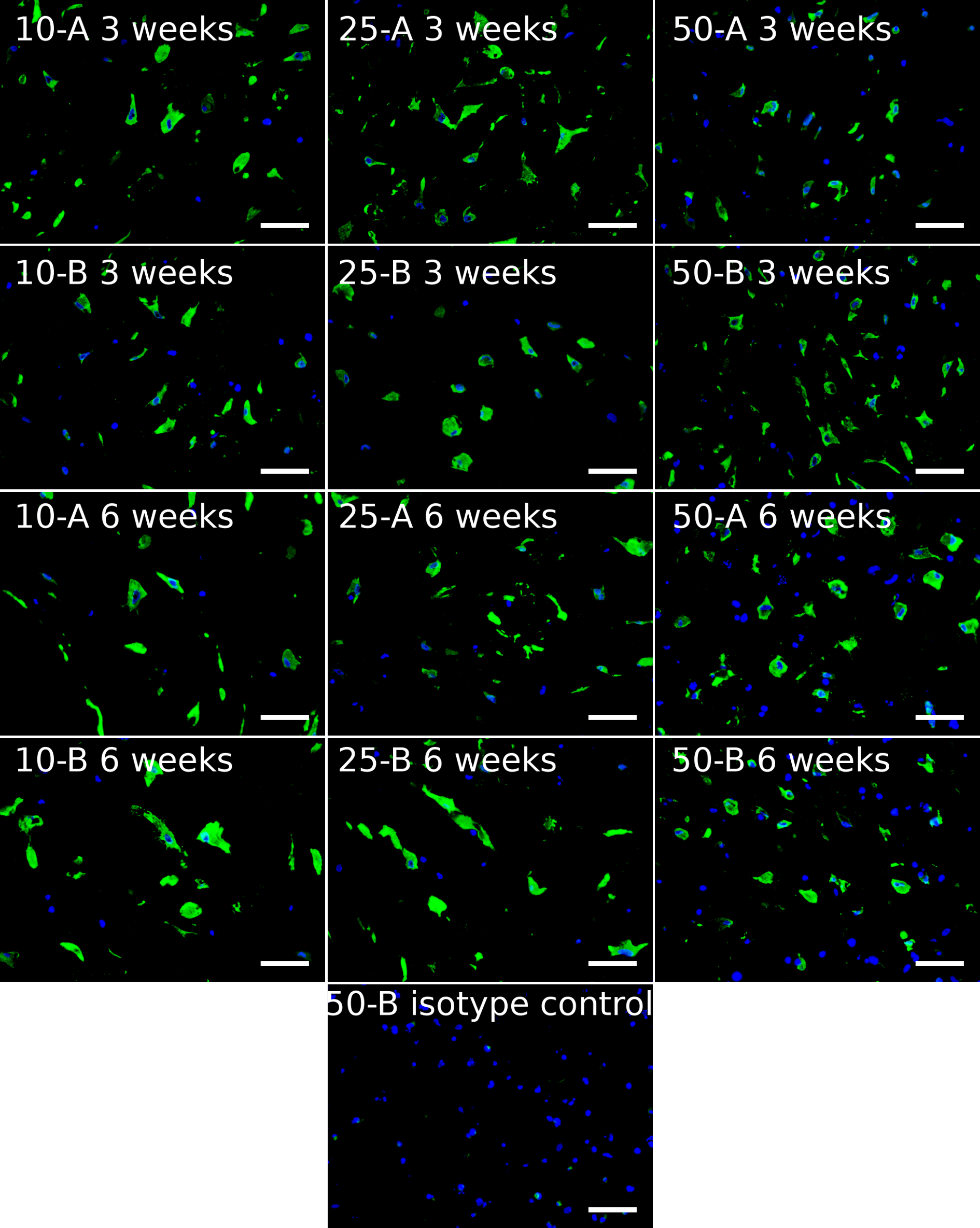

Supplement: Figure S9 — IHC staining of BMP-1 (green) and nuclear stain (blue) after three and six weeks of chondrogenic differentiation in alginate. Scale bars are 50 µm. (TIF) [file pone.0091662.s009.tif]

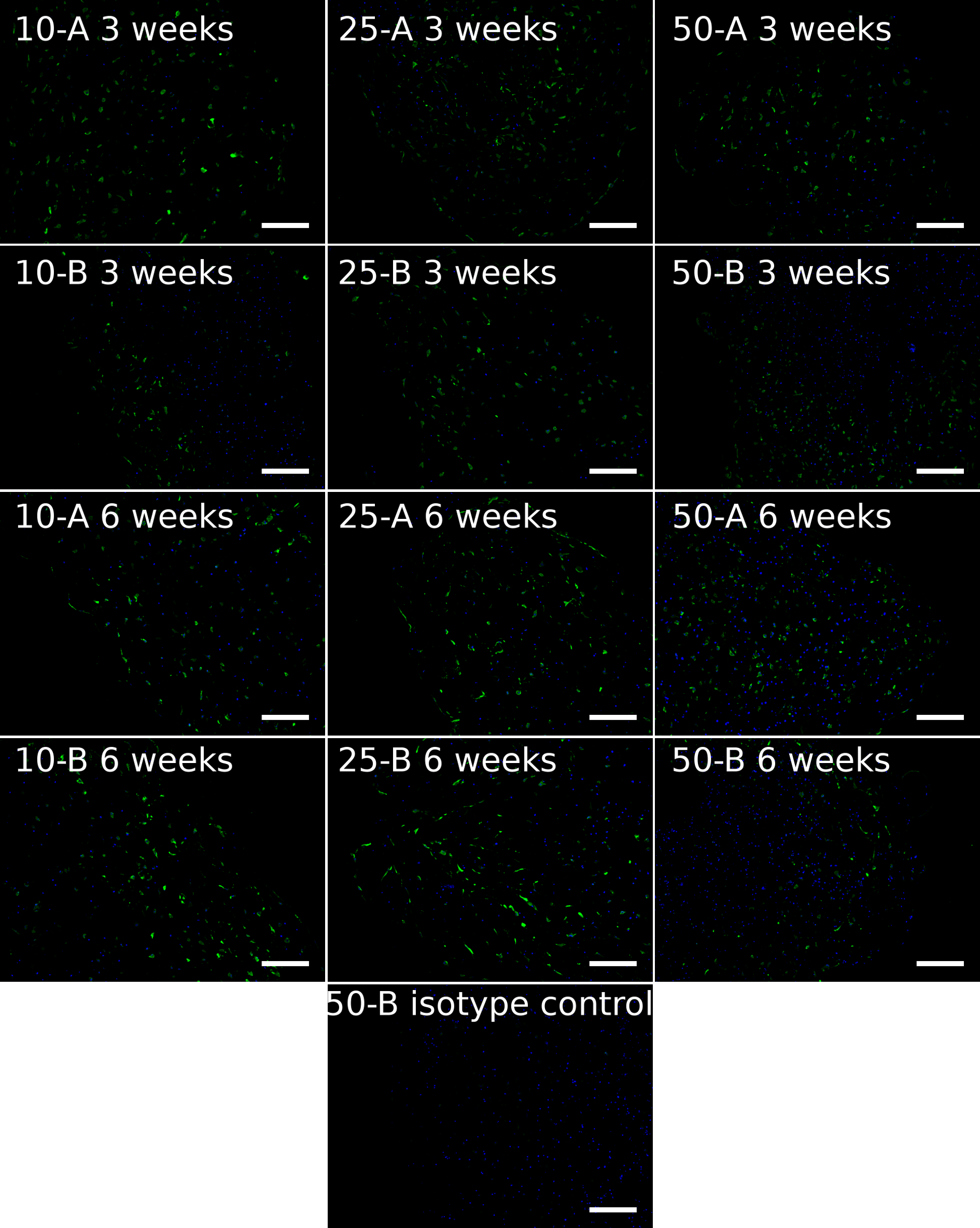

Supplement: Figure S10 — IHC staining of BMP-1 (green) and nuclear stain (blue) after three and six weeks of chondrogenic differentiation in alginate. Scale bars are 200 µm. (TIF) [file pone.0091662.s010.tif]
