# Supplementary figures and images for: Correction: Biochemical and Structural Characterization of Neocartilage Formed by Mesenchymal Stem Cells in Alginate Hydrogels
Source: PLoS One. 2014 Jun 19;9(6):e101096. doi: 10.1371/journal.pone.0101096 (PMC4063972; doi:10.1371/journal.pone.0101096)

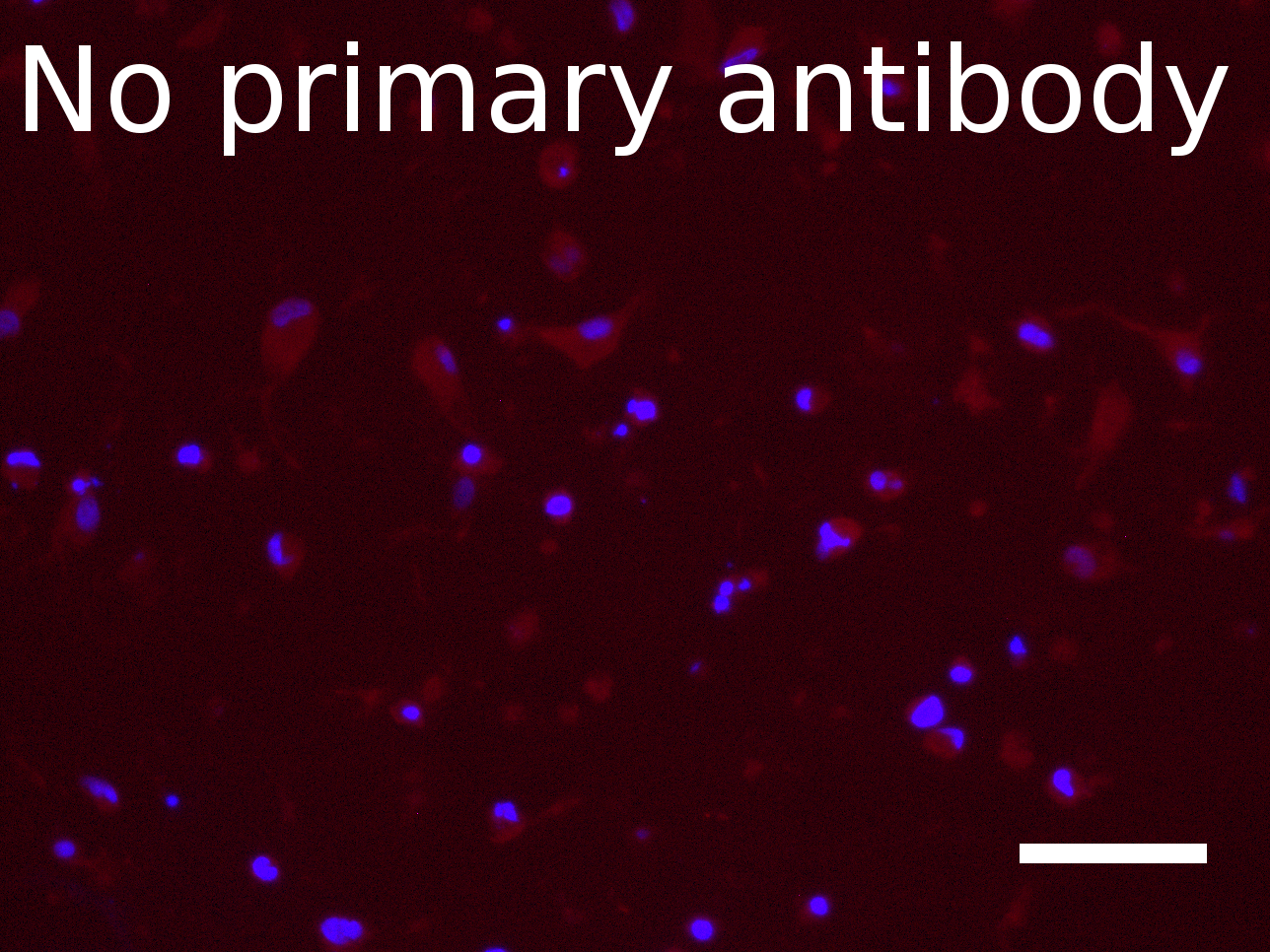

Supplement: Figure S6 — Negative control for type II procollagen omitting primary antibody. The image was captured using the same settings and the same post-processing as for the type II procollagen staining shown in Figure 2 in the article. Scale bar is 50 µm. (TIF) [file pone.0101096.s001.pdf]

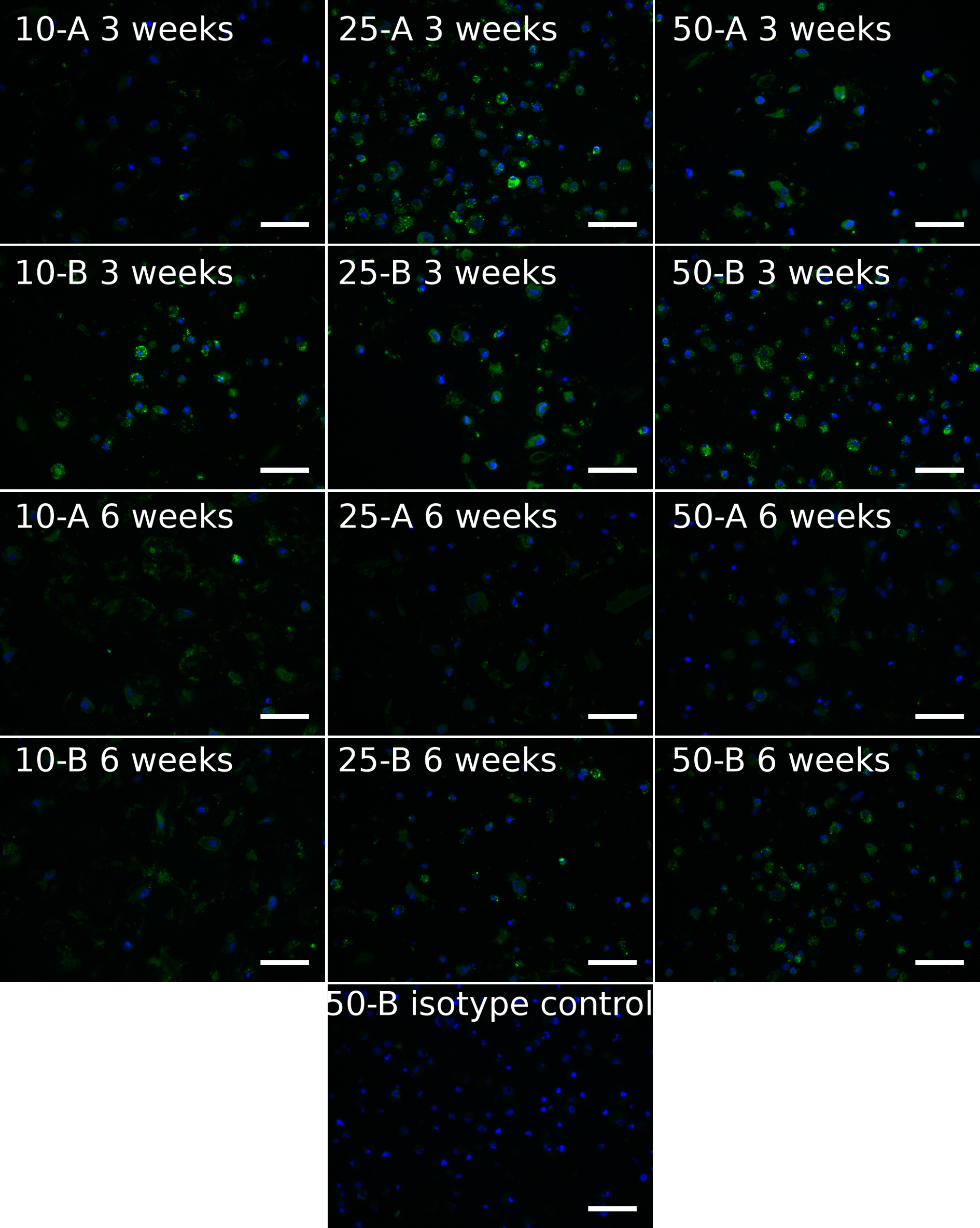

Supplement: Figure S7 — IHC staining of ADAMTS-2 (green) and nuclear stain (blue) after three and six weeks of chondrogenic differentiation in alginate. Scale bars are 50 µm. (TIF) [file pone.0101096.s002.pdf]

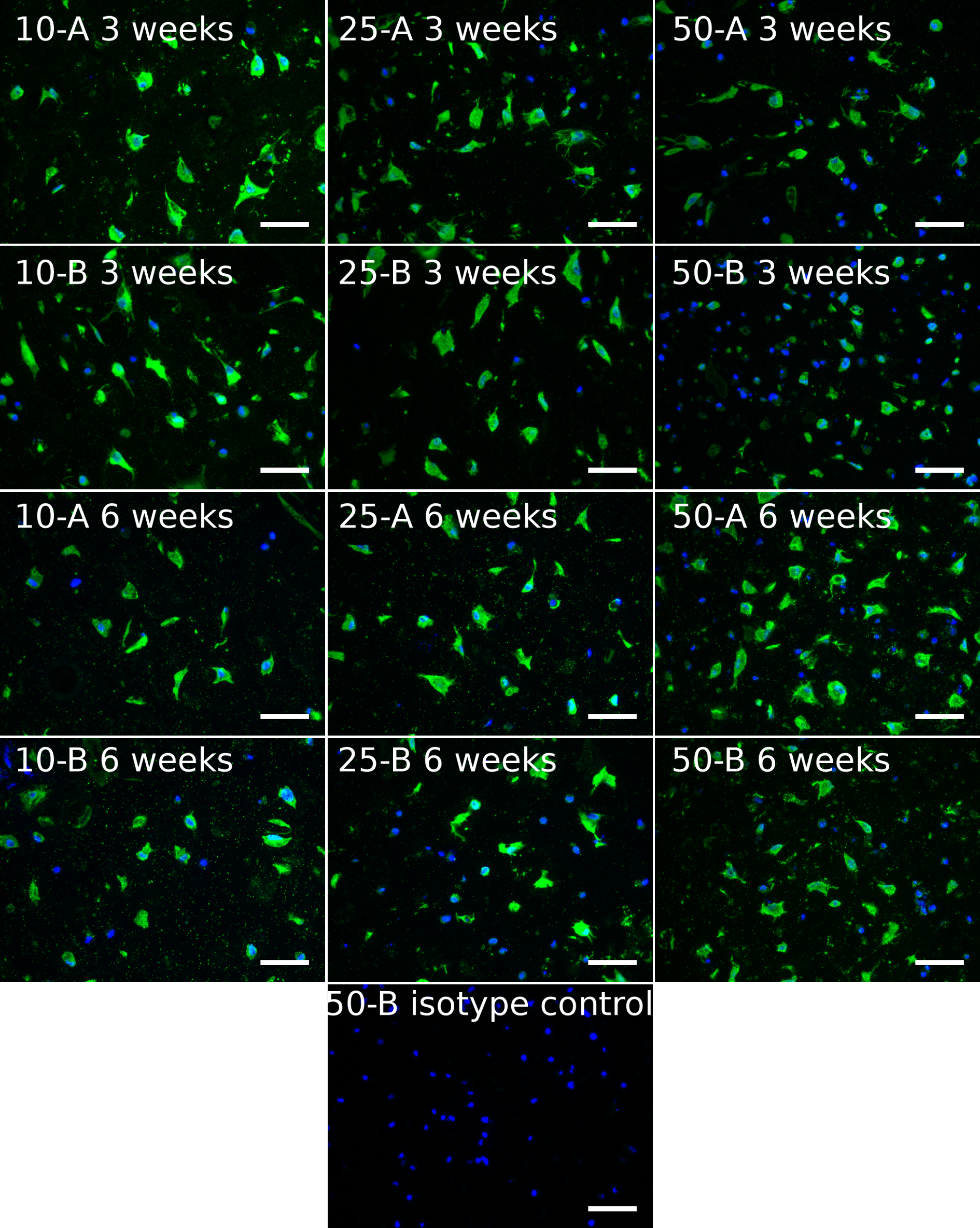

Supplement: Figure S8 — IHC staining of ADAMTS-3 (green) and nuclear stain (blue) after three and six weeks of chondrogenic differentiation in alginate. Scale bars are 50 µm. (TIF) [file pone.0101096.s003.pdf]
